# A Surrogate Modelling Approach Based on Nonlinear Dimension Reduction for Uncertainty Quantification in Groundwater Flow Models

**DOI:** 10.1007/s11242-018-1065-7

**Published:** 2018-05-25

**Authors:** C. Gadd, W. Xing, M. Mousavi Nezhad, A. A. Shah

**Affiliations:** School of Engineering, University of Warwick, Coventry, CV47AL UK

**Keywords:** Groundwater flow models, Uncertainty quantification, Surrogate model, Karhunen–Loève expansion, Manifold learning

## Abstract

In this paper, we develop a surrogate modelling approach for capturing the output field (e.g. the pressure head) from groundwater flow models involving a stochastic input field (e.g. the hydraulic conductivity). We use a Karhunen–Loève expansion for a log-normally distributed input field and apply manifold learning (local tangent space alignment) to perform Gaussian process Bayesian inference using Hamiltonian Monte Carlo in an abstract feature space, yielding outputs for arbitrary unseen inputs. We also develop a framework for forward uncertainty quantification in such problems, including analytical approximations of the mean of the marginalized distribution (with respect to the inputs). To sample from the distribution, we present Monte Carlo approach. Two examples are presented to demonstrate the accuracy of our approach: a Darcy flow model with contaminant transport in 2-d and a Richards equation model in 3-d.

## Introduction

Groundwater contamination, caused by landfills, wastewater seepage, hazardous chemical spillage, dumping of toxic substances or discharge from industrial processes (Karatzas 2017), is a major concern for both public and environmental health. Understanding the mechanisms and predicting the transport of contaminants through soils is therefore an important topic in groundwater flow modelling.

The control of groundwater quality relies on knowledge of the transport of chemicals to the groundwater through soil. The efficacy of remedial treatment and management of contaminated land depends on the accuracy of models used for the simulation of flow and solute transport. Modelling and simulation of hydraulic phenomena in soil are, however, hampered by the complex and heterogeneous nature of soils, as well as the broad range of influential factors involved. A number of simplified models have been developed to describe the small-scale physical, chemical (Boi et al. 2009; Foo and Hameed 2009; Vomvoris and Gelhar 1990), and biological mechanisms (Schfer et al. 1998; Barry et al. 2002) that affect unsaturated flow and contaminant transport.

A current challenge in modelling solute transport in soils lies in characterizing and quantifying the uncertainties engendered by the natural heterogeneity of the soil. Such uncertainty can be vital for decision-making. Despite strong evidence from field-scale observations and experimental studies in relation to the effects of soil heterogeneity on the transport of contaminants (Al-Tabbaa et al. 2000; Kristensen et al. 2010), relatively few numerical models incorporate the effects of this uncertainty (Feyen et al. 1998; Aly and Peralta 1999; Sreekanth and Datta 2011a, 2014; Herckenrath et al. 2011).

Monte Carlo (MC) sampling is the default method for investigating uncertainties in a system (e.g. propagating uncertainty in the inputs), including in the context of groundwater flow modelling (Fu and Gomez-Hernandez 2009; Paleologos et al. 2006; Kourakos and Harter 2014; Maxwell et al. 2007; Herckenrath et al. 2011). MC estimates are extracted from multiple runs of the model using different realizations of the inputs, sampled from some distribution. While convergence is guaranteed as the number of runs increases, the slow rate of convergence demands (typically) a few thousand runs in order to extract reliable estimates of the statistics. If the model is computationally expensive, such a brute-force approach can be extremely time-consuming or perhaps even infeasible (Maxwell et al. 2007). Analytical stochastic methods have also been employed (Gelhar and Axness 1983; Gelhar 1986). Such methods can be useful for conceptual understanding of the transport process but are not applicable to practical scenarios.

Such limitations and shortcomings could be resolved in theory by using surrogate models (also known as metamodels, emulators or simply surrogates) in place of the complex numerical codes; that is, computationally efficient approximations of the codes based on data-driven or reduced-order model (ROM) approaches. Surrogate models have been used in a limited number of groundwater flow modelling problems (Aly and Peralta 1999; Bhattacharjya and Datta 2005; Kourakos and Mantoglou 2009; Sreekanth and Datta 2011b; Ataie-Ashtiani et al. 2014) (we refer to Razavi et al. 2012; Ketabchi and Ataie-Ashtiani 2015 for reviews of the topic) and are typically based on artificial neural networks (ANNs) for approximating a small number of outputs within an optimization task. For example, Bhattacharjya and Datta used an ANN to approximate the salt concentration in pumped water at 8 pumping wells for 3 different times, in order to maximize the total withdrawal of water from a coastal aquifer while limiting the salt concentration (Bhattacharjya and Datta 2005). Similarly, Kourakos and Mantoglou used an ANN model to optimize 34 well pumping rates in a coastal aquifer (Kourakos and Mantoglou 2009).

Another popular surrogate modelling approach is the stochastic collocation method (Babuška et al. 2007) in which the approximate response is constrained to a subspace, typically spanned by a generalized Polynomial Chaos basis (Xiu and Karniadakis 2002). The coefficients in this basis are approximated via a collocation scheme. While these schemes yield good convergence rates, they scale poorly with the number of collocation points (Rajabi et al. 2015). Although sparse grid methods based on the Smolyak algorithm (Smolyak 1963) help to alleviate the increased computational burden, the resulting schemes are still severely limited by the input space dimensionality and tend to perform poorly with limited observations (Xiu and Hesthaven 2005; Xiu 2007; Nobile et al. 2008; Ma and Zabaras 2009).

When data are scarce, we may turn to statistical Bayesian approaches such as Gaussian process (GP) regression. GPs are stochastic processes used for inferring nonlinear and latent functions. They are defined as a families of normally distributed random variables, indexed in this case by the input variable(s). GPs were first used for surrogate models in the seminal papers of Currin et al. (1988) and Sacks et al. (1989). The first applications of GP surrogate models to uncertainty quantification can be found in  O’Hagan and Kingman (1978). Kernel methods such as GP models are well-established tools for analysing the relationships between input data and corresponding outputs of complex functions. Kernels encapsulate the properties of functions in a computationally efficient manner and provide flexibility in terms of model complexity (the functions used to approximate the target function) though variation of the functional form and parameters of the kernel.

GPs excel when data are scarce since they make a priori assumptions with regard to the relationship between data points. Comparatively, ANNs make fewer a priori assumptions and as a result require much larger data sets; they are, therefore, infrequently used for uncertainty quantification tasks. In the context of groundwater flow, very few applications of GPs can be found (Bau and Mayer 2006; Hemker et al. 2008; Borgonovo et al. 2012), the most likely explanations for which are the difficulty in implementing multioutput GP models and the lack of available information on, and software for GP modelling in comparison with ANNs. Existing applications again deal with low-dimensional outputs; e.g. in Bau and Mayer (2006), the authors use a GP model to learn 4 well extraction rates for a pump-and-treat optimization problem.

Our aim in this paper is to develop a surrogate model for the values of a field variable in a groundwater flow model, e.g. the pressure, pressure head or flow velocity, at a high number of points in the spatial domain, in order to propagate uncertainty in a stochastic field input, e.g. the hydraulic conductivity. In such cases, simplified covariance structures (Conti and O’Hagan 2010) for the output space (response surface) or dimensionality reduction for the input and/or output space can be used. In Higdon et al. (2008) Higdon et al. use principal component analysis (PCA) to perform linear, non-probabilistic dimensionality reduction on the response in order to render a GP model tractable (independent learning of a small number of PCA coefficients). Such linear approaches (PCA, multidimensional scaling, factor analysis) are applicable only when data lie in or near a linear subspace of the output space.

For more complex response surfaces, manifold learning (nonlinear dimensionality reduction) can be employed, using, for example, kernel principal component analysis (kPCA), diffusion maps (Xing et al. 2016 or isomaps Xing et al. 2015). In contrast, kPCA was used to perform nonlinear, non-probabilistic dimensionality reduction of the input space in Ma and Zabaras (2011). This can useful when the input space is generated from observations (experimental data), but when the form is specified we can use linear dimension reduction methods such as the Karhunen–Loève (KL) expansion (Wong 1971).

In this paper we use manifold learning in the form of local tangent space alignment (LTSA) (Zhang and Zha 2004) to perform Bayesian inference (GP regression/emulation with Markov Chain Monte Carlo) in an abstract feature space and use an inverse (pre-image) map to obtain the output field at a finite number of points for an arbitrary input. In contrast to diffusion maps, isomaps and kPCA, LTSA is a local method in that it approximates points on the manifold on localized regions (patches), rather than directly seeking a global basis for the feature space. This can potentially provide more accurate results, although this is of course dependent upon the sampling methodology for the points and the quality of the reconstruction mapping.

The aforementioned approach is combined with a Karhunen–Loève expansion for a log-normally distributed input field and a framework for UQ is developed. We derive analytical forms for the output distribution by pushing the feature space Gaussian distribution through a locally linear reconstruction map. Additionally, we derive analytical estimates of the moments of the predictive distribution via approximate marginalization of the stochastic input. To sample from the hyperparameter and signal precision posteriors, we employ a Hamiltonian Monte Carlo scheme and use MC sampling to approximately marginalize the stochastic input distribution. The accuracy of the approach is demonstrated via two examples: a linear, steady-state Darcy’s Law with a contaminant mass balance in a 2-d domain (aquifer) and a time-dependent Richards equation evaluated at a fixed time in a 3-d domain. In both cases we consider a stochastic hydraulic conductivity input.

The rest of the paper is organized as follows. In Sect. 2 we provide a detailed problem statement and outline the proposed solution. In Sect. 3 we outline LTSA, and in Sect. 4 we outline GP regression. In Sect. 5 we provide full details of the coupling of the methods and we demonstrate how the approach can be used to perform UQ tasks. In Sect. 6 we present the examples and discuss the results.

## Problem Statement

Consider a well-defined steady-state partial differential equation (PDE) with a scalar, isotropic random field input (e.g. a permeability or hydraulic conductivity), and a response (output) consisting of a scalar field, e.g. pressure head, concentration or flow velocity. We may generalize our approach to multiple or vector fields but in order to simplify the presentation we focus on a single scalar field. We can also apply the method we develop to dynamic problems by focusing on the spatial field at a given fixed time (the second example we present). For an arbitrary input field realization, solutions to the PDE are found using a numerical code (simulator, or solver) on a spatial mesh with $${k_{y}} $$ fixed degrees of freedom, e.g. grid points in a finite difference grid, control volume centres in a finite volume mesh or spatial nodes in a finite element mesh combined with a nodal basis.

We denote the input field by $K(\mathbf {x})$, where $\mathbf {x}\in \mathcal{R}\subset \mathbb {R}^{d}$, $d\in \{1,2,3\}$ denotes a spatial location and the notation makes explicit the spatial dependence. The model output (a scalar field) is denoted by $u(\mathbf {x};K)$, i.e. it is a function of $\mathbf {x}$ that is parameterized by $K(\mathbf {x})$. The random input $K(\mathbf {x})$ is defined on the whole of $\mathcal{R}$ and therefore requires a discrete (finite-dimensional) approximation in order to obtain a numerical solution. Let $\mathbf {x}_k\in \mathcal{R}^d$, $k=1,\ldots ,{k_{y}}$ be a set of nodes or grid points and suppose that the simulator yields discrete approximations $\{u_k = u(\mathbf {x}_k;K)\}_{k=1}^{{k_{y}} }$ of the output field $u(\mathbf {x};K)$ in each run. Our goal is to approximate these simulator outputs for an arbitrary *K*.

### Input Model: Karhunen–Loève Expansion

Let $(\Omega , \mathcal{F}, \mathbb {P})$ be a probability space, with sample space $\Omega$, event space $\mathcal{F}$ and probability measure $\mathbb {P}$. We can explicitly signify the randomness of the input by writing $K(\mathbf {x},\omega )$, where $\omega \in \Omega$. For simplicity, and where it will not cause confusion, we suppress the dependence on $\omega$ (the same applies to other random processes). We assume that $K(\mathbf {x})$ is log-normal (to avoid unphysical, i.e. negative, realizations), so is of the form $K(\mathbf {x},\omega )=\exp (Z(\mathbf {x},\omega ))$, where $Z(\mathbf {x},\omega )$ is a normally distributed field (a GP^1^ indexed by $\mathbf {x}$). For each $\mathbf {x}\in \mathcal{R}$, $Z(\mathbf {x},\cdot ):\Omega \rightarrow \mathbb {R}$ is a random variable defined on the (common) probability space $(\Omega , \mathcal{F}, \mathbb {P})$. For a fixed $\omega \in \Omega$, $Z(\cdot ,\omega ):\mathcal{R}\rightarrow \mathbb {R}$ is a deterministic function of $\mathbf {x}$ called a realization or sample path of the process. The mean and covariance functions of $Z(\mathbf {x},\omega )$ are defined as:

1 $$\begin{aligned} \begin{aligned} m_Z(\mathbf {x})&\displaystyle =\mathbb {E}[Z(\mathbf {x},\omega )]=\int _{\Omega }Z(\mathbf {x},\omega )\text {d}\mathbb {P}(\omega ), \\ c_Z(\mathbf {x}, \mathbf {x}')&\displaystyle =\mathbb {E}\left[ (Z(\mathbf {x},\omega )-m_Z(\mathbf {x}))(Z(\mathbf {x}',\omega )-m_Z(\mathbf {x}'))\right] , \end{aligned} \end{aligned}$$

respectively, in which $\mathbb {E}[\cdot ]$ is the expectation operator. Given the covariance and mean functions for $Z(\mathbf {x},\omega )$, the most widely used finite-dimensional approximation is based on a Karhunen–Loève (KL) expansion (Wong 1971). Assume that $Z(\mathbf {x},\omega )$ is mean-square continuous ($\lim _{\delta \mathbf {x}\rightarrow \mathbf{0}}\mathbb {E}[(Z(\mathbf {x}+\delta \mathbf {x},\omega )-Z(\mathbf {x},\omega ))^2]=0$) and that $Z(\mathbf {x},\omega )\in L^2(\mathcal{R}\times \Omega )$ ($\int _\mathcal{R}\mathbb {E}[|Z(\mathbf {x},\omega )|^2]<\infty$), and is thus a second-order process. The KL theorem states that we may expresses $Z(\mathbf {x},\omega )$ as a linear combination of deterministic $L^2(\mathcal{R})$-orthonormal functions $w_j(\mathbf {x})$, with random $L^2(\Omega )$-orthonormal coefficients $\xi _j(\omega )$:

2 $$\begin{aligned} Z(\mathbf {x},\omega )=m_Z(\mathbf {x})+\sum _{j=1}^\infty \sqrt{\lambda _j} \xi _j(\omega )w_j(\mathbf {x}), \end{aligned}$$

where $\lambda _1\ge \lambda _2\ge \cdots >0$ and $\{w_j(\mathbf {x})\}_{j=1}^\infty$ are, respectively, the eigenvalues and eigenfunctions of an integral operator with kernel $c_Z(\mathbf {x}, \mathbf {x}')$:

3 $$\begin{aligned} \int _\mathcal{R} c_Z(\mathbf {x}, \mathbf {x}') w_j(\mathbf {x}')\text {d}\mathbf {x}'=\lambda _jw_j(\mathbf {x}). \end{aligned}$$

The random coefficients are given by:

4 $$\begin{aligned} \xi _j(\omega )=\frac{1}{\sqrt{\lambda _j}}\int _\mathcal{R}(Z(\mathbf {x}',\omega )-m_Z(\mathbf {x}'))w_j(\mathbf {x}')\text {d}\mathbf {x}', \end{aligned}$$

and are independent, standard normal ($\xi _j\sim \mathcal{N}(0,1)$), with $\mathbb {V}\text{ ar }(\sqrt{\lambda _j}\xi _j(\omega ))=\lambda _j$, where $\mathbb {V}\text{ ar }(\cdot )$ denotes the variance operator. Here and throughout, $\mathcal{N}(\cdot ,\cdot )$ denotes a normal distribution, in which the first argument is the mean (mean vector) and the second is the variance (covariance matrix).

The sum (2) can be truncated by virtue of the decay in the eigenvalues for increasing *j*. Discretizing the eigenvalue problem (3) using finite differencing at the nodes $\mathbf {x}_k\in \mathcal{R}$, $k=1,\ldots ,{k_{y}}$, assuming that they are uniformly distributed, leads to an eigenvalue problem for the covariance matrix $\mathbf {C}=\left[ c_Z(\mathbf {x}_k, \mathbf {x}_j)\right] _{k,j=1}^{{k_{y}} }$:

5 $$\begin{aligned} \mathbf{C}_Z\mathbf{w}_j=\lambda _j\mathbf{w}_j, \end{aligned}$$

where the *k*th component $w_{j,k}$ of $\mathbf{w}_j\in \mathbb {R}^{{k_{y}} }$, $j=1,\ldots ,{k_{y}}$, is equivalent to the evaluation of eigenfunction $w_j$ at the node $\mathbf {x}_k$, $k=1,\ldots ,{k_{y}}$. Defining the random vector $\mathbf{Z} := (Z(\mathbf {x}_1),\ldots ,Z(\mathbf {x}_{{k_{y}} }))^T:\Omega \rightarrow \mathbb {R}^{{k_{y}} }$, we can write:

6 $$\begin{aligned} \mathbf{Z}=\mathbf {m}_Z+ \sum _{j=1}^{{k_{y}} } \sqrt{\lambda _j} \xi _j(\omega )\mathbf{w}_j, \end{aligned}$$

where $\mathbf {m}_Z = (m_Z(\mathbf {x}_1),\ldots ,m_Z(\mathbf {x}_{{k_{y}} }))^T$ and $\xi _j\sim \mathcal{N}(0,1)$ are independent random variables (note that we have kept the notation $\xi _j$ and $\lambda _j$ used in the continuous case in order to avoid notational clutter). This provides discrete realizations of $Z(\mathbf {x},\omega )$, and the expansion in (6) can be truncated by virtue of the decay in $\lambda _j$ for some ${k_{\xi }} <{k_{y}}$, chosen so that the generalized variance satisfies $\sum _{j=1}^{{k_{\xi }} } \sqrt{\lambda _j}/ \sum _{j=1}^{{k_{y}} } \sqrt{\lambda _j} >\vartheta$ for some specified tolerance $0<\vartheta <1$. We can then obtain discrete realizations $\mathbf{K}=(K_1,\ldots ,K_{{k_{y}} })^T$ of $K(\mathbf {x},\omega )$ via:

7 $$\begin{aligned} K_k=K(\mathbf {x}_k,\omega )=\exp \left( m_Z(\mathbf {x}_k)+ \sum _{j=1}^{{k_{\xi }} } \sqrt{\lambda _j} \xi _j(\omega )w_{j,k}\right) . \end{aligned}$$

The discrete input $\mathbf{K}$ can then replaced by the random vector defined by $\varvec{\xi }=(\xi _1,\ldots ,\xi _{{k_{\xi }} })^T\sim \mathcal{N}(\mathbf{0},\mathbf{I})$, the coefficients of which are independent standard normal. We may then write $u(\mathbf {x}_k;\varvec{\xi })$ for the KL approximation to $u(\mathbf {x}_k;K)$, at the nodes $\{\mathbf {x}_k\}_{k=1}^{{k_{y}} }$.

We note that different methods, including different quadrature rules or the use of projection schemes and Nystrom methods (Wan and Karniadakis 2006) can be used to solve the eigenvalue problem (3), all of which lead to a generalized eigenvalue problem in place of (5) (Betz et al. 2014). For example, if the finite element method is used, we may express the eigenfunctions as $w_j(\mathbf {x})=\sum _k l_{j,k}\psi _k$ in terms of the finite element basis $\{\psi _k\}_{k=1}^{{k_{y}} }$ and perform a Galerkin projection of (3) onto $\text{ span }(\psi _1,\ldots ,\psi _{{k_{y}} })$ to yield a generalized eigenvalue problem for $\{\lambda _j\}_{j=1}^{{k_{y}} }$ and the undetermined coefficients $\{l_{j,k}\}_{j,k=1}^{{k_{y}} }$ (Ghanem and Spanos 2003).

### Statement of the Surrogate Model Problem

The simulator can now be considered as a mapping $\varvec{\eta }:\mathcal {X}\rightarrow \mathcal {Y}$ (assumed to be continuous and injective), where $\varvec{\xi }\in \mathcal {X}\subset \mathbb {R}^{{k_{\xi }} }$ is the permissible *input space* and $\mathbf {y}\in \mathcal {Y}\subset \mathbb {R}^{{k_{y}} }$ is the permissible *output space* or *response surface* consisting of the discrete field:

8 $$\begin{aligned} \mathbf{y}=\varvec{\eta }(\varvec{\xi }):=(u(\mathbf {x}_1;\varvec{\xi }),\ldots ,u(\mathbf {x}_{{k_{y}} };\varvec{\xi }))^T. \end{aligned}$$

Our aim is to develop a surrogate to make fast, online predictions of $\varvec{\eta }(\varvec{\xi })$, using *training data* from a limited number of solver runs at the *design points*$\varvec{\xi }_n$, $n=1,\ldots ,N$. The training data can be expressed compactly as a matrix $\mathbf {Y}=\left[ \mathbf {y}_1, \dots , \mathbf {y}_N\right] ^T\in \mathbb {R}^{N\times {k_{y}} }$ and we can define $\pmb {\Xi }=\left[ \varvec{\xi }_1, \dots , \varvec{\xi }_N\right] ^T\in \mathbb {R}^{N\times {k_{\xi }} }$. The data set is thus $\mathcal {D}' = \{\pmb {\Xi },\mathbf {Y}\}$.

The high dimensionalities of the (original) input and output spaces pose great challenges for surrogate model development. The input space dimensionality can be reduced as described above. The intrinsic dimensionality of the output space is significantly lower than $${k_{y}} $$ by virtue of correlations between outputs for different inputs, as well as physical constraints imposed by the simulator. This suggests that we treat $$\mathcal{Y}$$ as a manifold and use manifold learning/dimensionality reduction to perform Bayesian inference on a low-dimensional (feature) space $$\mathcal{F}$$ that is locally homeomorphic to $$\mathcal{Y}$$. Below we introduce the manifold learning method employed, before recasting the emulation problem as one of inference in the feature space, together with a pre-image (inverse) mapping to obtain solutions in $$\mathcal{Y}$$ for arbitrary inputs $$\varvec{\xi }$$.

## Dimensionality Reduction and Manifold Learning: Feature Space Representations

Roughly speaking, a $${k_{z}} $$-dimensional manifold $$\mathcal {Y}$$ is a set for which all points can be parameterized by $${k_{z}} $$ independent variables. A parameterization is called a coordinate system (or a chart) and it is not necessarily the case that a single coordinate system can describe the entire manifold. To characterize the manifold in such cases, we can introduce overlapping patches, each with its own system of (non-unique) coordinates.

Formally speaking, a smooth $${k_{z}} $$-manifold is defined as a topological space $$\mathcal {Y}$$ that is equipped with a maximal open cover $$\{U_{\alpha }\}_{\alpha \in \Gamma }$$ consisting of coordinate neighbourhoods (or patches) $$U_{\alpha }$$, together with a collection of homeomorphisms (coordinate charts) $$\phi _{\alpha }: U_{\alpha }\rightarrow \phi _{\alpha }(U_{\alpha }) \subset \mathbb {R}^{k_{z}} $$ onto open subsets $$\phi _{\alpha }(U_{\alpha }) \subset \mathbb {R}^{k_{z}} $$ such that $$\phi _{\alpha }(U_{\alpha }\cap U_{\beta })$$ and $$\phi _{\beta }(U_{\alpha }\cap U_{\beta })$$ are open in $$\mathbb {R}^{k_{z}} $$; we say that $$\phi _{\alpha }$$ and $$\phi _{\beta }$$ are compatible. Moreover, the transition maps defining a change of coordinates $$\phi _{\beta } \circ \phi _{\alpha }^{-1}$$ are diffeomorphisms for all $$\alpha ,\beta \in \Gamma $$.

Let $$\mathcal {A}=\{(U_{\alpha },\phi _{\alpha })\}_{\alpha \in \Gamma }$$ be an atlas on $$\mathcal {Y}$$ ($$\{U_{\alpha }\}_{\alpha \in \Gamma }$$ is a cover and the $$\{\phi _{\alpha }\}_{\alpha \in \Gamma }$$ are pairwise compatible). Two smooth curves $$\gamma _0,\gamma _1:\mathbb {R}\rightarrow \mathcal {Y}$$ are called $$\mathbf {y}$$-equivalent at a point $$\mathbf {y}\in \mathcal {Y}$$ if for every $$\alpha \in \Gamma $$ with $$\mathbf {y}\in U_{\alpha }$$, we have $$\gamma _0(0)=\gamma _1(0)=\mathbf {y}$$ and furthermore $$(\text {d}/\text {d}t)|_{t=0}\phi _{\alpha }(\gamma _0(t))=(\text {d}/\text {d}t)|_{t=0}\phi _{\alpha }(\gamma _1(t))$$. With this equivalence relation, the equivalence class of a smooth curve $$\gamma $$ with $$\gamma (0)=v$$ is denoted $$[\gamma ]_p$$ and the *tangent space*$$T_\mathbf {y}\mathcal {Y}$$ of $$\mathcal {Y}$$ at $$\mathbf {y}$$ is the set of equivalence classes $$\{[\gamma ]_p:\gamma (0)=\mathbf {y}\}$$. The tangent space is a $${k_{z}} $$-dimensional vector space, which is seen more clearly by identifying $$T_\mathbf {y}\mathcal {Y}$$ with the set of all derivations at $$\mathbf {y}$$ [linear maps from $$C^\infty (\mathcal {Y})$$ to $$\mathbb {R}$$ satisfying the derivation (Liebnitz) property].

We assume that the output space $\mathcal {Y}\supset \mathbf {Y}$ is a manifold of dimension ${k_{z}} \ll {k_{y}}$ embedded in $\mathbb {R}^{k_{y}}$. Representations of points in $\mathcal {Y}$ and corresponding representations in the feature or latent space $\mathcal{F}\subset \mathbb {R}^{k_{z}}$ can be related by some smooth and *unknown* function $\mathbf {f}: \mathcal{F} \rightarrow \mathcal {Y}$. *Manifold learning* is concerned with the reconstruction of $\mathbf {f}$ and its inverse given data points on the manifold, whereas *dimensionality reduction* is concerned with the representation of given points in $\mathcal {Y}$ by corresponding points in the feature space $\mathcal{F}$. Here we are interested primarily in dimensionality reduction and use *Local Tangent Space Alignment* (LTSA) (Zhang and Zha 2004). The tangent space at a point $\mathbf {y}$ provides a low-dimensional linear approximation of points in a neighbourhood of $\mathbf {y}$. We can approximate each point $\mathbf {y}$ in a data set using a basis for $T_\mathbf {y}\mathcal {Y}$ and use these approximations to find low-dimensional representations in a global coordinate system, by aligning the tangent spaces using local affine transformations (Zhang and Zha 2004). We note that this assumes the existence of a single chart (homeomorphism) $\mathbf {f}^{-1}$.

Consider a noise-free model in which the data $\mathbf {Y}$ are generated by the smooth function $\mathbf {f}$ defined above:

9 $$\begin{aligned} \mathbf {y}= \mathbf {f}(\mathbf {z}) = \left( f_1 (\mathbf {z}),\dots , f_{k_{y}} (\mathbf {z})\right) ^T, \end{aligned}$$

where $\mathbf {z}=(z_1,\ldots ,z_{k_{z}} )^T\in \mathcal{F}$ is a latent/feature vector (i.e. the low-dimensional representation of the point $\mathbf {y}$). Under the assumption that $\mathbf {f}$ is smooth, it can be approximated using a first-order Taylor expansion in a neighbourhood $\Omega (\mathbf {z})$ of a point $\mathbf {z}$: $\mathbf {f}(\widehat{\mathbf {z}}) = \mathbf {f}(\mathbf {z}) + \mathbf{J}_\mathbf {f}(\mathbf {z})\cdot (\widehat{\mathbf {z}}-\mathbf {z}) + \mathcal {O}(\Vert \widehat{\mathbf {z}}-\mathbf {z}\Vert ^2), \;\forall \widehat{\mathbf {z}}\in \Omega (\mathbf {z})$, where $\mathbf{J}_\mathbf {f}(\mathbf {z}) \in \mathbb {R}^{{k_{y}} \times {k_{z}} }$ is the Jacobi matrix of $\mathbf {f}$ at $\mathbf {z}$, the *i*, *j*th entry of which is $\partial f_i/\partial z_j$. Here and throughout, $||\cdot ||$ denotes a standard Euclidean norm.

A basis for the tangent space $$T_\mathbf {y}\mathcal{Y}$$ of $$\mathcal{Y}$$ (a $${k_{z}} $$-dimensional linear subspace of $$\mathbb {R}^{k_{y}} $$) at $$\mathbf {y}=\mathbf {f}(\mathbf {z})$$ is given by the span of the column vectors of $$\mathbf{J}_\mathbf {f}$$. The vector $$\widehat{\mathbf {z}}-\mathbf {z}$$ then gives the coordinate of $$\mathbf {f}\left( \widehat{\mathbf {z}}\right) $$ in the affine subspace $$\mathbf {f}(\mathbf {z}) + T_\mathbf {y}\mathcal{Y}$$. $$\mathbf{J}_\mathbf {f}$$ cannot be computed explicitly without knowledge of $$\mathbf {f}$$. Suppose we can express $$T_\mathbf {y}\mathcal{Y}$$ in terms of a matrix $$\mathbf{Q}_\mathbf {z}$$, the columns of which form an orthonormal basis for $$T_\mathbf {y}\mathcal{Y}$$:

10 $$\begin{aligned} \mathbf{J}_f(\mathbf {z})\cdot \left( \widehat{\mathbf {z}}-\mathbf {z}\right) = \mathbf{Q}_\mathbf {z}\pmb {\pi }^*_\mathbf {z}, \end{aligned}$$

where $\pmb {\pi }^*_\mathbf {z}= \mathbf{Q}_\mathbf {z}^T \mathbf{J}_\mathbf {f}(\mathbf {z}) \cdot \left( \widehat{\mathbf {z}}-\mathbf {z}\right) \equiv \mathbf{P}_\mathbf {z}\left( \widehat{\mathbf {z}}-\mathbf {z}\right)$ is still unknown. Combining Eq. (10) with the Taylor expansion, we can, however, find an approximation of $\pmb {\pi }^*_\mathbf {z}$ consisting of an orthogonal projection of $\mathbf {f}\left( \widehat{\mathbf {z}}\right) - \mathbf {f}(\mathbf {z})$ onto $T_\mathbf {y}\mathcal{Y}$:

11 $$\begin{aligned} \pmb {\pi }_\mathbf {z}\equiv \mathbf{Q}_\mathbf {z}^T \left( \mathbf {f}\left( \widehat{\mathbf {z}}\right) -\mathbf {f}(\mathbf {z})\right) = \pmb {\pi }^*_\mathbf {z}+ \mathcal {O}\left( \Vert \widehat{\mathbf {z}}-\mathbf {z}\Vert ^2\right) , \end{aligned}$$

provided that the basis $\mathbf{Q}_\mathbf {z}$ is known for each $\mathbf {z}$. Truncating this expansion, the global coordinate $\mathbf {z}$ then satisfies:

12 $$\begin{aligned} \int \int _{\Omega (\mathbf {z})} \Vert \mathbf{P}_\mathbf {z}\left( \widehat{\mathbf {z}}-\mathbf {z}\right) - \pmb {\pi }_\mathbf {z}\Vert \text {d}\widehat{\mathbf {z}} \approx 0. \end{aligned}$$

If the Jacobian is of full column rank, we can find a local affine transformation:

13 $$\begin{aligned} \widehat{\mathbf {z}} - \mathbf {z}\approx \mathbf{P}^{-1}_\mathbf {z}\pmb {\pi }_\mathbf {z}\equiv \mathbf{L}_\mathbf {z}\pmb {\pi }_\mathbf {z}. \end{aligned}$$

The transformation $\mathbf{L}_\mathbf {z}$ aligns the local coordinate with the global coordinate $\widehat{\mathbf {z}}-\mathbf {z}$ for $f(\widehat{\mathbf {z}})$. We then find the global coordinate $\widehat{\mathbf {z}}$ and affine transformation $\mathbf{L}_\mathbf {z}$ by minimizing $\int \int _{\Omega (\mathbf {z})} \Vert \widehat{\mathbf {z}}-\mathbf {z}- \mathbf{L}_\mathbf {z}\pmb {\pi }_\mathbf {z}\Vert \text {d}\widehat{\mathbf {z}}$.

We note that the orthogonal basis $\mathbf{Q}_\mathbf {z}$ for each tangent space is still unknown. Consider a data set $\mathbf {y}_n$, $n=1,\ldots ,N$, sampled with noise $\epsilon _n$, $n=1,\ldots ,N$, from the underlying nonlinear manifold:

14 $$\begin{aligned} \mathbf {y}_n = \mathbf {f}(\mathbf {z}_n)+\epsilon _n. \end{aligned}$$

For any $$\mathbf {y}_n$$, let $$\mathbf {Y}_n=[\mathbf {y}_{n_1}\ldots \mathbf {y}_{n_P}]$$ be the matrix containing the *P* nearest neighbours, including $$\mathbf {y}_n$$, where distances are measured using the standard Euclidean metric. The best $${k_{z}} $$-dimensional local affine subspace approximation for the points in $$\mathbf {Y}_n$$ is given by:

15 $$\begin{aligned} \mathop {\mathrm {arg\,min}}_{\mathbf {y},\pmb {\Pi },\mathbf{Q}} \sum _{k=1}^{P} \Vert \mathbf {y}_{n_k} - \left( \mathbf {y}+\mathbf{Q}\pmb {\pi }_k\right) \Vert ^2_2&= \mathop {\mathrm {arg\,min}}_{\mathbf {y},\pmb {\Pi },\mathbf{Q}} \Vert \mathbf {Y}_n - (\mathbf {y}\mathbf{e}^T+\mathbf{Q}\pmb {\Pi }) \Vert ^2_2, \end{aligned}$$

where the orthonormal matrix $$\mathbf{Q}$$ has $${k_{z}} $$ columns, $$\pmb {\Pi } = \left[ \pmb {\pi }_1 \dots \pmb {\pi }_P \right] $$ and $$\mathbf{e}$$ is a vector of all ones. The optimal $$\mathbf {y}$$ is given by the mean of $$\{\mathbf {y}_{n_k}\}_k$$, denoted $$\bar{\mathbf {y}}_n$$, and the optimal $$\mathbf{Q}$$ is given by $$\mathbf{Q}_n$$, the columns of which are the $${k_{z}} $$ left singular vectors of $$\mathbf {Y}_n\left( \mathbf{I}-\mathbf{e}{} \mathbf{e}^T/P\right) $$ corresponding to the $${k_{z}} $$ largest singular values. Lastly, $$\pmb {\Pi }$$ is given by $$\pmb {\Pi }_n$$:

16 $$\begin{aligned} \pmb {\Pi }_n = \mathbf{Q}_n^T \mathbf {Y}_n\left( \mathbf{I}-\frac{1}{P}{} \mathbf{e}\mathbf{e}^T\right) = \left[ \pmb {\pi }^{\left( i\right) }_1,\dots ,\pmb {\pi }^{\left( i\right) }_K\right] , \end{aligned}$$

where $$\pmb {\pi }^{\left( i\right) }_k = \mathbf{Q}_n^T\left( \mathbf {y}_{n_k}-\bar{\mathbf {y}}_n\right) $$. Consequently:

17 $$\begin{aligned} \mathbf {y}_{n_k} = \bar{\mathbf {y}}_n + \mathbf{Q}_n\pmb {\pi }^{\left( l\right) }_k + \varphi ^{\left( l\right) }_k, \end{aligned}$$

where $\varphi ^{\left( l\right) }_k = \left( I-\mathbf{Q}_n \mathbf{Q}_n^T\right) \left( \mathbf {y}_{n_k} - \bar{\mathbf {y}}_n\right)$ is the reconstruction error. Having minimized the local reconstruction error, we would like to find the global coordinates $\mathbf {Z}= \left[ \mathbf {z}_1\ldots \mathbf {z}_N \right] \in \mathbb {R}^{{k_{z}} \times N}$, corresponding to data points $\mathbf {Y}$, given the local coordinates $\pmb {\pi }^{\left( l\right) }_k$. The global coordinates $\mathbf {z}_{n_k}$ of the corresponding points $\mathbf {y}_{n_k}$ are chosen to respect the local geometry as determined by the $\pmb {\pi }^{\left( l\right) }_k$:

18 $$\begin{aligned} \begin{aligned} \mathbf {z}_{n_k}&= \bar{\mathbf {z}}_n + \mathbf{L}_n\pmb {\pi }^{\left( l\right) }_k + \epsilon ^{\left( l\right) }_k, \quad k=1,\ldots ,P, \;l=1,\ldots ,N,\\ \mathbf {Z}_n&= \frac{1}{P}\mathbf {Z}_n \mathbf{e}{} \mathbf{e}^T + \mathbf{L}_n\pmb {\Pi }_n + \mathbf{E}_n, \end{aligned} \end{aligned}$$

where $\bar{\mathbf {z}}_n$ is the mean of $\{\mathbf {z}_{n_k}\}_k$, $\mathbf {Z}_n = [\mathbf {z}_{n_1} \ldots \mathbf {z}_{n_P}]$ and $\mathbf{E}_n = [\epsilon ^{\left( l\right) }_1 \ldots \epsilon ^{\left( l\right) }_P]$, given by $\mathbf{E}_n = \mathbf {Z}_n (\mathbf{I} - \mathbf{e}{} \mathbf{e}^T/P) -\mathbf{L}_n\pmb {\Pi }_n$. We find the latent points and local affine transformations $\mathbf{L}_n$ that minimize the local reconstruction error $\Vert \mathbf{E}_n \Vert _F$, in which $||\cdot ||_F$ denotes a Frobenius norm. The optimal $\mathbf{L}_n$ are given by $\mathbf{L}_n = \mathbf {Z}_n(\mathbf{I}-\mathbf{e}{} \mathbf{e}^T/P)\pmb {\Pi }_n^+$, and consequently the errors are given by $\mathbf{E}_n = \mathbf {Z}_n(\mathbf{I}-\mathbf{e}\mathbf{e}^T/P)(\mathbf{I}-\pmb {\Pi }_n^+\pmb {\Pi }_n)$, where $\pmb {\Pi }_n^+$ is the Moor–Penrose pseudo-inverse of $\pmb {\Pi }_n$. We define a 0-1 selection matrix $\mathbf{S}_n\in \mathbb {R}^{N\times P}$ such that $\mathbf {Z}\mathbf{S}_n = \mathbf {Z}_n$. The global coordinates can then be selected according to a minimization of the overall reconstruction error:

19 $$\begin{aligned} \mathop {\mathrm {arg\,min}}_{\mathbf {Z}:\mathbf {Z}^T \mathbf {Z}=\mathbf{I}} \sum _n \Vert \mathbf{E}_n \Vert _F^2 = \mathop {\mathrm {arg\,min}}_{\mathbf {Z}:\mathbf {Z}^T \mathbf {Z}=\mathbf{I}} \Vert \mathbf {Z}\mathbf {S} \mathbf {W} \Vert _F^2, \end{aligned}$$

where $\mathbf {S} = \left[ \mathbf{S}_1 \ldots \mathbf{S}_N\right]$, and $\mathbf {W} = {\text {diag}}\left( \mathbf{W}_1,\dots , \mathbf{W}_N\right)$, in which $\mathbf{W}_n = (\mathbf{I} - \mathbf{e}{} \mathbf{e}^T/P)(\mathbf{I}-\pmb {\Pi }_n^+ \pmb {\Pi }_n)$. The constraint $\mathbf {Z}^T \mathbf {Z}=\mathbf{I}$ ensures that the solutions are unique. The vector $\mathbf{e}$ is an eigenvector of $\mathbf {B}\equiv \mathbf {S} \mathbf {W}\mathbf {W}^T \mathbf {S}^T\in \mathbb {R}^{N\times N}$ corresponding to a zero eigenvalue. Arranging the eigenvalues in increasing order, the optimal $\mathbf {Z}$ is given by $\mathbf {Z}'=[\pmb {\zeta }_2 \ldots \pmb {\zeta }_{{k_{z}} +1}]^T$, where $\pmb {\zeta }_2, \ldots , \pmb {\zeta }_{{k_{z}} +1} \in \mathbb {R}^N$ are the eigenvectors of $\mathbf {B}$ corresponding to the $\left( {k_{z}} +1\right) ^\text {st}$ smallest eigenvalues excluding the first (zero) eigenvalue. This defines a map $\mathbf {f}^-:\mathbf {y}\mapsto \mathbf {z}$, $\mathbf {z}=\mathbf {f}^-(\mathbf {y})$ that approximates $\mathbf {f}^{-1}:\mathcal{Y}\rightarrow \mathcal{F}$*for the given data points*:

20 $$\begin{aligned} \mathbf {z}_n=\mathbf {f}^{-1}(\mathbf {y}_n)\approx \mathbf {f}^-(\mathbf {y}_n)=\mathbf {z}'_{n,:}. \end{aligned}$$

where $\mathbf {z}'_{n,:}$ is the *n*th column of $\mathbf {Z}'$.

Fixing the number of neighbours assumes that the manifold has a certain smoothness, while using the same number of neighbours for every tangent space assumes a global smoothness. These assumptions may result in inaccurate predictions, in which case we can use adaptive algorithms (Zou and Zhu 2011; Zhang et al. 2012; Wei et al. 2008). Similar adaptations can be made for other issues, such as robustness in the presence of noise (Zhan and Yin 2011).

We remark that LTSA is a nonparametric technique, in that an explicit form of $\mathbf {f}$ is not available. This means that the *out-of-sample* problem does not have a parametric (explicit) solution. In other words, application of LTSA (the map $\mathbf {f}^-$) to a point that was not in the data set can only be achieved by rerunning the entire algorithm with an updated data set that appends the new point. Nonparametric solutions to the out-of-sample problem have been developed, and one that is applicable to LTSA can be found in Li et al. (2005).

If we map points $\mathbf {y}\in \mathcal{Y}$ to $\mathcal{F}$ using $\mathbf {f}^-$ and perform inference in $\mathcal{F}$, an approximation of $\mathbf {f}$ is required in order to make predictions in the physical space $\mathcal{Y}$. This is referred to as the *pre-image* problem in manifold learning methods: given a point in the low-dimensional space, find a mapping to the original space (manifold). We outline an approximation of the pre-image map in the next section.

### Pre-image Problem: Reconstruction of Points in the Manifold $\mathcal {Y}$

Given a point $\mathbf {z}\in \mathcal{F}$ in latent space, we require the corresponding point in the original physical space $\mathbf {y}\in \mathcal {Y}$. Let $\mathbf {z}_k$ be the neighbour nearest to $\mathbf {z}$. According to Eq. 18:

21 $$\begin{aligned} \pmb {\pi }_*^{\left( k\right) } = \mathbf{L}_k^{-1}\left( \mathbf {z}- \bar{\mathbf {z}}_k\right) - \mathbf{L}_k^{-1}\epsilon _*^{\left( k\right) }. \end{aligned}$$

By Eq. 17 we can also define:

22 $$\begin{aligned} \mathbf {y}= \bar{\mathbf {y}}_k + \mathbf{Q}_k\pmb {\pi }_*^{\left( k\right) } + \varphi ^{\left( k\right) }_*. \end{aligned}$$

Consequently, we have the following approximate pre-image mapping $\hat{\mathbf {f}}: \mathcal {F} \rightarrow \mathcal {Y}$ (approximation of $\mathbf {f}$):

23 $$\begin{aligned} \begin{aligned} \mathbf {y}=\mathbf {f}(\mathbf {z})\approx \hat{\mathbf {f}}(\mathbf {z})&= \bar{\mathbf {y}}_k +\mathbf{Q}_k \left( \mathbf{L}^{-1}_k\left( \mathbf {z}- \bar{\mathbf {z}}_k\right) - \mathbf{L}^{-1}_k\epsilon _*^{\left( k\right) }\right) + \varphi ^{\left( k\right) }_*\\&= \bar{\mathbf {y}}_k + \mathbf{Q}_k\mathbf{L}_k^{-1}\left( \mathbf {z}-\bar{\mathbf {z}}_k\right) + \mathcal {E}, \end{aligned} \end{aligned}$$

where $k=\mathop {\mathrm {arg\,min}}_n\Vert \mathbf {z}- \mathbf {z}_n\Vert$ and $\mathcal {E}=-\mathbf{Q}_k \mathbf{L}_k^{-1}\epsilon _*^{(k)}+\varphi _*^{(k)}$ incorporates the error terms.

## Gaussian Process Emulation in Feature Space

In Sect. 2.2, the surrogate model problem was defined as one of approximating the simulator mapping $\varvec{\eta }:\mathcal {X}\rightarrow \mathcal {Y}$ given the data set $\mathcal {D}' = \{\pmb {\Xi },\mathbf {Y}\}$ derived from runs of the simulator at selected design points $\{\varvec{\xi }_n\}_{n=1}^N$. We can instead consider the simulator as a mapping $\varvec{\eta }_\mathcal{F}\equiv \mathbf {f}^{-1}\circ \varvec{\eta }:\mathcal{X}\rightarrow \mathcal{F}$ from the input space to the feature space, i.e. $\varvec{\eta }_\mathcal{F}(\cdot )= \mathbf {f}^{-1}(\varvec{\eta }(\cdot ))$. Application of LTSA to points on the manifold approximates this mapping with $\mathbf {f}^-\approx \mathbf {f}^{-1}$. The original data set $\mathcal {D}' = \{\pmb {\Xi },\mathbf {Y}\}$ is replaced by the equivalent data set $\mathcal {D}=\{\pmb {\Xi },\mathbf {Z}\}$ or $\mathcal{D}=\left\{ (\varvec{\xi }_n,\,\mathbf {z}_n)\right\} _{n=1}^N$, where $\mathbf {z}_n=\mathbf {f}^-(\mathbf {y}_n)\approx \mathbf {f}^{-1}(\mathbf {y}_n)=\mathbf {f}^{-1}(\varvec{\eta }(\varvec{\xi }_n))=\varvec{\eta }_\mathcal{F}(\varvec{\xi }_n)$, and our aim is now to approximate the mapping $\varvec{\eta }_\mathcal{F}(\cdot )$. Returning a general point $\mathbf {z}=\varvec{\eta }_\mathcal{F}(\varvec{\xi })$ to the corresponding point $\mathbf {y}$ in the space $\mathcal{Y}$ is discussed in the next section.

In this work, a GP model is used to infer the mapping $\varvec{\eta }_\mathcal{F}:\varvec{\xi }\mapsto \mathbf {z}$ by treating it as a realization of a (Gaussian) stochastic process indexed by the inputs $\varvec{\xi }$. Specifically, we learn each component of $\mathbf {z}$ separately (assuming independence) using a *scalar* GP model. Here and throughout, $\mathcal{GP}(\cdot ,\cdot )$ denotes a GP, in which the first argument is the mean function and the second is the covariance (kernel) function.

Let $$z_{n,i}$$, $$i=1,\ldots ,{k_{z}} $$, denote the *i*th component of $$\mathbf {z}_n$$, $$n=1,\ldots ,N$$. The probabilistic model is $$z_{n,i}=h_i(\varvec{\xi }_n)+\eta _{n,i}$$, in which the signal noise distribution is $$p(\eta _{n,i})=\mathcal{N} (0,\beta _i^{-1})$$$$\forall n$$, where $$\beta _i$$ is the precision. We assume independent GP priors $$h_i(\varvec{\xi })\sim \mathcal{GP}(0,c_h(\varvec{\xi },\varvec{\xi }';\varvec{\theta }_i))$$, where $$c_h(\varvec{\xi },\varvec{\xi }';\varvec{\theta }_i)$$ is the kernel function (of the same form across *i*) in which $$\varvec{\theta }_i$$ is a vector of hyperparameters pertaining to component *i*. The latent functions $$h_i(\varvec{\xi })$$, $$i=1,\ldots ,{k_{z}} $$, can be thought of as independent draws from the GP. Using the notation $$h_{n,i}=h_i(\varvec{\xi }_n)$$ we can define a matrix $$\mathbf{H}\in \mathbb {R}^{N\times {k_{z}} }$$ with columns $$\mathbf{h}_{:,i}=(h_{1,i},\ldots ,h_{N,i})^T$$ ($$\mathbf {z}_{:,i}$$ similarly defines the vector of the *i*th features across all samples). By the independence assumption:

24 $$\begin{aligned} p(\mathbf{H}|\varvec{\Xi },\varvec{\Theta })=\prod _{i=1}^{{k_{z}} }p(\mathbf{h}_{:,i}|\varvec{\Xi },\varvec{\theta }_i), \end{aligned}$$

where $\varvec{\Theta }=[\varvec{\theta }_1\ldots \varvec{\theta }_{{k_{z}} }]$. By the properties of GPs, we have $p(\mathbf{h}_{:,i}|\varvec{\Xi },\varvec{\theta }_i)=\mathcal {N} (\mathbf {0}, \mathbf{C}_i)$, where $\mathbf{C}_i\in \mathbf {R}^{N\times N}$ is a kernel matrix, the *n*, *m*th entry of which is $c_h(\varvec{\xi }_n,\varvec{\xi }_m;\varvec{\theta }_i)$. Thus:

25 $$\begin{aligned} \begin{aligned} p(\mathbf {Z}| \varvec{\Xi },\varvec{\Theta }, \pmb {\beta })&= \int \prod _{i=1}^{{k_{z}} }\prod _{n=1}^{N}p(z_{n,i} | h_{n,i},\beta _i) p(\mathbf{h}_{:,i} | \varvec{\Xi }, \varvec{\theta }_i ) \text {d}{} \mathbf{H} \\&= \prod _{i=1}^{{k_{z}} } \mathcal {N}(\mathbf {0}, \mathbf{C}_i + \beta _i^{-1} \mathbf {I}), \end{aligned} \end{aligned}$$

where $p(\mathbf {z}_{:,i}|\mathbf{h}_{:,i},\beta _i) = \mathcal {N}(\mathbf{h}_{:,i},\beta _i^{-1}\mathbf {I})$ by virtue of the noise model, and $\pmb {\beta }=(\beta _1,\ldots ,\beta _{{k_{z}} })^T$.

We place gamma priors on all hyperparameters $\varvec{\theta }_i$ and signal noise precisions $\beta _i$. The parameterization of these priors is determined through an initial maximum likelihood run. We choose these parameters such that the mean is equal to the maximum likelihood value, and so that we obtain an appropriate variance. Let $\mathbf {z}\in \mathcal{F}$ be the feature vector corresponding to the test (new) input $\varvec{\xi }$. The predictive distribution for the *i*th component $z_i$ of $\mathbf {z}$ ($i=1,\ldots ,{k_{z}}$) is given by:

26 $$\begin{aligned} \begin{aligned} p\left( z_i |\varvec{\xi }, \mathcal {D},\varvec{\theta }_i,\beta _i \right)&= \mathcal {N}\left( \mu _i(\varvec{\xi }), \sigma _i^2(\varvec{\xi })\right) ,\\ \mu _i(\varvec{\xi })&= \mathbf {c}_h(\varvec{\xi },\pmb {\Xi };\varvec{\theta }_i)^T \left( \mathbf{C}_i+\beta _i^{-1}{} \mathbf{I}\right) ^{-1}\mathbf {z}_{:,i}, \\ \sigma _i^2(\varvec{\xi })&= c_h\left( \varvec{\xi },\varvec{\xi };\varvec{\theta }_i\right) - \mathbf {c}_h(\varvec{\xi },\pmb {\Xi };\varvec{\theta }_i)^T\left( \mathbf {C}_i + \beta _i^{-1} \mathbf{I}\right) ^{-1} \mathbf {c}_h(\varvec{\xi },\pmb {\Xi };\varvec{\theta }_i), \end{aligned} \end{aligned}$$

where $\mathbf {c}_h(\varvec{\xi },\pmb {\Xi };\varvec{\theta }_i)=(c_h(\varvec{\xi }_1,\varvec{\xi };\varvec{\theta }_i),\ldots ,c_h(\varvec{\xi }_N,\varvec{\xi };\varvec{\theta }_i))^T\in \mathbb {R}^N$ is the cross-covariance between $\mathbf {z}$ and $\mathbf {z}_n$, $n=1,\ldots ,N$. Thus, the latent variable GP prediction is distributed as:

27 $$\begin{aligned} \begin{aligned} p(\mathbf {z}|\varvec{\xi },\mathcal {D},\varvec{\Theta },\pmb {\beta })&= \mathcal {N}(\pmb {\mu }_{\mathbf {z}}(\varvec{\xi }),\Sigma _{\mathbf {z}}(\varvec{\xi })),\\ \pmb {\mu }_{\mathbf {z}}(\varvec{\xi })&=(\mu _1(\varvec{\xi }),\ldots ,\mu _{{k_{z}} }(\varvec{\xi }))^T,\\ \Sigma _\mathbf {z}(\varvec{\xi })&=\text{ diag }(\sigma _1^2(\varvec{\xi }),\ldots ,\sigma _{{k_{z}} }^2(\varvec{\xi })), \end{aligned} \end{aligned}$$

where the components of $\pmb {\mu }_{\mathbf {z}}(\varvec{\xi })\in \mathcal{F}$ are given by the second of Eqs. (26) and $\Sigma _\mathbf {z}(\varvec{\xi }) \in \mathbb {R}^{{k_{z}} \times {k_{z}} }$ is a diagonal covariance matrix, in which the *i*th diagonal element corresponds to the predictive variance given by the third of Eqs. (26), while the off-diagonal elements are zero due to the assumption of independent GPs across *i*.

### Sampling Hyperparameter Posterior with Hybrid Monte Carlo

We explore the hyperparameter posterior distributions using a hybrid Monte Carlo (HMC) scheme. HMC is a Metropolis method that uses gradient information. It exploits Hamiltonian dynamics to explore state spaces based on the likelihood probability, and consequently limits the random walk behaviour. The Hamiltonian is defined as an energy function in terms of a position vector $\mathbf {q}(t)$ and a momentum vector $\mathbf {p}(t)$ at time *t* (unrelated to the time component in the solver): $H\left( \mathbf {q}(t),\mathbf {p}(t)\right) =E_U(\mathbf {q}(t)) + E_K(\mathbf {p}(t))$, where $E_U(\mathbf {q})$ is the potential energy and $E_K(\mathbf {p})$ is the kinetic energy, the sum of which is constant. The evolution of this system is then defined by the partial derivatives of the Hamiltonian:

28 $$\begin{aligned} \begin{aligned} \frac{d\mathbf {p}}{dt} = - \frac{\partial H}{\partial \mathbf {q}}, \quad \frac{d\mathbf {q}}{dt} = + \frac{\partial H}{\partial \mathbf {p}}. \end{aligned} \end{aligned}$$

We define the potential energy as the negative log likelihood with an additive constant *C*, chosen for convenience: $E_U(\mathbf {q}(t)) = - \log \left( \text {likelihood}\left( \mathbf {q}(t)\right) \right) - \log \left( \text {prior}\left( \mathbf {q}\left( t\right) \right) \right)$. Furthermore, following convention we define the kinetic energy as:

29 $$\begin{aligned} E_K(\mathbf {p}(t)) = \frac{1}{2} \mathbf {p}(t) \mathbf{M}_K^{-1} \mathbf {p}(t), \end{aligned}$$

where $\mathbf{M}_K$ is a symmetric, positive definite mass matrix, chosen to be a scalar multiple of the identity matrix. With this choice, the potential energy is the negative log probability density of a multivariate Gaussian distribution with covariance $\mathbf{M}_K$ and matches the classical definition of potential energy.

## Predictions

The physical models we consider have an unknown, stochastic input (e.g. the hydraulic conductivity). This represents a lack of knowledge of the input, which induces a random variable response (e.g. the pressure head). Quantifying the distribution over the response is referred to as a pushforward or *forward problem*. The *pushforward measure* is the distribution over the response, or quantity of interest derived from the response.^2^ Based on the methods of the preceding sections, we now present an emulation framework for interrogating the pushforward measure (the response distribution). We begin by describing in the next section how a single realization of the random variable response may be obtained given a single realization of the stochastic input. In Sect. 5.2, we then discuss how to quantify the pushforward measure (extract relevant statistics of the response).

### Outputs Conditioned on Inputs

Due to the nature of the emulator, the prediction of a point $\mathbf {z}\in \mathcal{F}$ is normally distributed. This distribution captures uncertainty in the predictions as a consequence of limited and noise corrupted data. A common challenge when using reduced dimensional representations is analytically propagating this distribution through a nonlinear, pre-image map [in this case $\hat{\mathbf {f}}:\mathcal{F}\ni \mathbf {z}\mapsto \mathbf {y}\in \mathcal {Y}$ defined by Eq. (23)] for a test input $\varvec{\xi }$.

Analytically propagating a distribution through a nonlinear mapping is often not feasible. Instead we could repeatedly sample from the feature space response distribution (over $\mathbf {z}\in \mathcal{F}$) and apply the pre-image map to find the distribution over the corresponding $\mathbf {y}\in \mathcal{Y}$. Examples that use this latter approach include kernel principal component analysis and Gaussian process latent variable models (in the latter case, approximations can be obtained using the projected process approximation). Since the manifold consists of aligned (tangent) hyperplanes, however, we are able to derive locally linear pre-image maps that can be used for mapping distributions defined on local tangent spaces. The latent variable GP prediction $\mathbf {z}$ is distributed according to Eq. (27). Restricting to a single tangent space, it is a straightforward task to push this distribution though Eq. (23) to obtain a normal distribution for the corresponding $\mathbf {y}\in \mathcal{Y}$:

30 $$\begin{aligned} \begin{aligned} p(\mathbf {y}|{\varvec{\xi }},\mathcal {D},\varvec{\Theta },\pmb {\beta })&= \mathcal {N}\left( \pmb {\mu }_{\mathbf {y}}(\varvec{\xi }),\Sigma _{\mathbf {y}}(\varvec{\xi })\right) , \\ \pmb {\mu }_{\mathbf {y}}(\varvec{\xi })&= \bar{\mathbf {y}}_k + \mathbf{Q}_k\mathbf{L}_k^{-1}\left( \pmb {\mu }_{\mathbf {z}}(\varvec{\xi }) - \bar{\mathbf {z}}_k\right) ,\\ \Sigma _{\mathbf {y}}(\varvec{\xi })&= \mathbf{Q}_k\mathbf{L}_k^{-1} \Sigma _{\mathbf {z}}(\varvec{\xi }) \left( \mathbf{Q}_k\mathbf{L}_k^{-1}\right) ^T, \end{aligned} \end{aligned}$$

where $k=\mathop {\mathrm {arg\,min}}\nolimits _n\Vert \pmb {\mu }_\mathbf {z}(\varvec{\xi }) - \mathbf {z}_n \Vert$, $\pmb {\mu }_{\mathbf {y}}(\varvec{\xi })\in \mathbb {R}^{{k_{y}} }$, and $\Sigma _{\mathbf {y}}(\varvec{\xi }) \in \mathbb {R}^{{k_{y}} \times {k_{y}} }$. This result is particularly useful for scenarios in which knowledge of the correlations between response features is required. Without this result we would require a large number of samples to estimate the covariance (tens of thousands). If, however, we are only interested in samples of the distribution (27), i.e. making predictions at specified inputs, then it is more memory efficient to sample from the predictive distribution (27) and use the pre-image map (23). When the support of this distribution is large, the accuracy of the local approximation breaks down and we must first sample the latent features before applying the pre-image map.

### Marginalizing the Stochastic Input

Having obtained a distribution over the response for a stochastic input realization, we now consider the problem of obtaining a distribution over the response marginalized over the stochastic input. We assume that the input is normally distributed:

31 $$\begin{aligned} p(\varvec{\xi })= \mathcal {N}\left( \pmb {\mu }_{\varvec{\xi }},\Sigma _{\varvec{\xi }}\right) , \end{aligned}$$

for some mean vector $\pmb {\mu }_{\varvec{\xi }}$ (equal to $\mathbf{0}$ in this case) and covariance matrix $\Sigma _{\varvec{\xi }}$ (equal to $\mathbf{I}$ in this case). We wish to evaluate:

32 $$\begin{aligned} \begin{aligned} p\left( \mathbf {y}|\mathcal {D},\varvec{\Theta },\pmb {\beta }\right) =\hat{\mathbf {f}}\left( p(\mathbf {z}|\mathcal {D},\varvec{\Theta },\pmb {\beta })\right)&= \hat{\mathbf {f}}\left( \int p(\mathbf {z}|\varvec{\xi }', \mathcal {D},\varvec{\Theta },\pmb {\beta })p(\varvec{\xi }') \text {d}\varvec{\xi }' \right) , \end{aligned} \end{aligned}$$

where $\hat{\mathbf {f}}$ is the (measurable) pre-image map and $p\left( \mathbf {z}|\varvec{\xi },\mathcal {D},\varvec{\Theta },\pmb {\beta }\right)$ is defined in Eq. (27). Since the input $\varvec{\xi }$ appears nonlinearly in the inverse of the $\mathbf {z}$ predictive distribution covariance $\sigma ^2(\varvec{\xi })$, we are unable to find a closed form solution to the integral in (32), i.e. the marginal distribution over $\mathbf {z}$. The moments of this marginal distribution can, on the other hand, be found analytically, although we will not know the family of distributions to which these moments belong.

Let us focus on the *i*th feature of $\mathbf {z}$. We wish to find the first two moments, i.e. the mean and variance, of the marginal distribution $p\left( z_i|\mathcal {D},\varvec{\theta }_i,\beta _i\right)$. We can then push these moments through the pre-image map to obtain analytical solutions. This can be repeated for each *i* by virtue of the independence assumption. To begin, $p\left( z_i|\mathcal {D},\varvec{\theta }_i,\beta _i\right)$ is approximated as a Gaussian with mean *m* and variance *v* (Girard and Murray-Smith 2003), which, from “Appendix A”, are given by:

33 $$\begin{aligned} \begin{aligned} m = \mathbb {E}_{\varvec{\xi }}\left[ \mathbf {c}_h(\varvec{\xi },\pmb {\Xi };\varvec{\theta }_i)\right] ^T \left( \mathbf{C}_i+\beta _i^{-1} \mathbf{I}\right) ^{-1}\mathbf {z}_{:,i} \end{aligned} \end{aligned}$$

and:

34 $$\begin{aligned} \begin{aligned} v =\,&\mathbb {E}_{\varvec{\xi }}\left[ c_h(\varvec{\xi },\varvec{\xi };\varvec{\theta }_i) \right] - m^2\\&-\left[ \left( \mathbf{C}_i+\beta _i^{-1} \mathbf{I}\right) ^{-1}-\left( \left( \mathbf{C}_i+\beta _i^{-1}\mathbf{I}\right) \mathbf {z}_{:,i}\right) ^2 \right] \mathbb {E}_{\varvec{\xi }}\left[ \mathbf {c}_h(\varvec{\xi },\pmb {\Xi };\varvec{\theta }_i)^T\mathbf {c}_h(\varvec{\xi },\pmb {\Xi };\varvec{\theta }_i) \right] . \end{aligned} \end{aligned}$$

where $\mathbb {E}_{\varvec{\xi }}[\cdot ]$ and $\mathbb {V}\text{ ar }_{\varvec{\xi }}(\cdot )$ are the expectation and variance with respect to ${\varvec{\xi }}$, respectively. Calculation of these moments involves expectations of the kernel with respect to the stochastic input distribution on the unknown and unobserved test inputs:

35 $$\begin{aligned} \begin{aligned} \mathbb {E}_{\varvec{\xi }}\left[ c_h(\varvec{\xi },\varvec{\xi };\varvec{\theta }_i) \right]&= \int c_h(\varvec{\xi }',\varvec{\xi }';\varvec{\theta }_i)p(\varvec{\xi }')\text {d}\varvec{\xi }',\\ \mathbb {E}_{\varvec{\xi }}\left[ \mathbf {c}_h(\varvec{\xi },\pmb {\Xi };\varvec{\theta }_i)\right]&= \int \mathbf {c}_h(\varvec{\xi }',\pmb {\Xi };\varvec{\theta }_i)p(\varvec{\xi }')\text {d}\varvec{\xi }',\\ \mathbb {E}_{\varvec{\xi }}\left[ \mathbf {c}_h(\varvec{\xi },\pmb {\Xi };\varvec{\theta }_i)^T \mathbf {c}_h(\varvec{\xi },\pmb {\Xi };\varvec{\theta }_i)\right]&= \int \mathbf {c}_h(\varvec{\xi }',\pmb {\Xi };\varvec{\theta }_i)^T\mathbf {c}_h(\varvec{\xi }',\pmb {\Xi };\varvec{\theta }_i) p(\varvec{\xi }')\text {d}\varvec{\xi }'. \end{aligned} \end{aligned}$$

The analytic tractability of these integrals is dependent upon the choice of kernel and stochastic input distribution. One example of a kernel is the commonly used squared exponential, for which the integrals are derived in “Appendix B”. Once calculated, the mean can be pushed through the local pre-image mapping (23). Since we expect that the variance, on the other hand, will span more than one tangent space, predictions of the variance using this method may be inaccurate.

Since we cannot sample from the approximate marginal of the analytical approach, further analysis requires MC to fully characterize the distribution (32). Again it is sufficient to demonstrate the procedure for a single latent (feature space) dimension *i*. Using MC we obtain a marginalized predictive distribution expressed as the sum of normally distributed random variables, which itself is non-Gaussian:

36 $$\begin{aligned} \begin{aligned} p(z_{\cdot ,i}|\mathcal {D},\varvec{\theta }_i,\beta _i)&= \int p(z_{\cdot ,i}|\varvec{\xi }',\mathcal {D},\varvec{\theta }_i,\beta _i)p(\varvec{\xi }') \text {d}\varvec{\xi }' \\&\simeq \frac{1}{Q}\sum _{q=1}^{Q} p(z_{\cdot ,i} |\varvec{\xi }^{(q)},\mathcal {D},\varvec{\theta }_i,\beta _i) \\&= \frac{1}{Q} \sum _{q=1}^{Q} \mathcal {N} (\mu (\varvec{\xi }^{(q)}),\sigma ^2(\varvec{\xi }^{(q)})), \end{aligned} \end{aligned}$$

where $\varvec{\xi }^{(q)}\sim \mathcal {N}(\pmb {\mu }_{\varvec{\xi }},\Sigma _{\varvec{\xi }})$, $\varvec{\theta }_i$ and $\beta _i$ are samples from the hyperparameter and signal noise posteriors (for the *i*th feature), and the approximation converges as $Q\rightarrow \infty$. Each sampled latent variable can then be pushed through the pre-image map. Latent variables found in this way are draws from the marginalized distribution $p\left( z_{\cdot ,i}|\mathcal {D},\varvec{\theta }_i,\beta _i\right)$ and we obtain multiple marginalized distributions [one for each $(\varvec{\theta }_i,\beta _i)$] from which we can estimate the statistics of the response. Algorithm 1 describes the procedure. Note that we use a $*$ superscript in order to avoid confusion between MC samples and training points. Each $\mathbf {Y}_i^*$ in Algorithm 1 can be interrogated to find any property of the pushforward measure (mean, standard deviation and higher-order moments). We can use kernel density estimation (KDE, also known as Parzen–Rosenblatt window) (Simonoff 1996) to approximate the pdf given a finite number of samples, or find the moments of the density. We use Gaussian kernel function with a suitably small bandwidth.
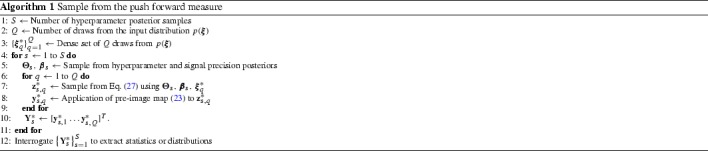


## Results and Discussion

We now assess the performance of the proposed method on two example partial differential equation problems: a Darcy flow problem with a contaminant mass balance, modelling steady-state groundwater flow in a 2-d porous medium; and Richards equation, modelling single-phase flow through a 3-d porous medium. As explained in Sect. 5, the analysis includes: (i) predictions that are conditioned on an input; and (ii) predictions that are marginalized over the stochastic input.

When making conditioned predictions, we use the conditional predictive distribution (30) for $\mathbf {y}$, or the distribution (27) for $\mathbf {z}$ in conjunction with the pre-image map (23). As explained in 4.1, we place a prior over the hyperparameters $\varvec{\Theta }$ and signal variances $\pmb {\beta }$ and use a HMC scheme to sample from the posterior distributions over these parameters. Each sample can be used to obtain a different normal predictive distribution, conditioned on an input. We are therefore able to see how the predictive mean and variance change with respect to the uncertainty in the GP parameters. In the results, we plot the expectation and standard deviation of first two predictive distribution moments.

For the forward UQ problem we marginalize the conditional predictive distributions over a stochastic input (Eq. 32) to obtain the pushforward measure (non analytically). We are able to analytically find the mean using (A2) and (A3) together with the pre-image map, or, using Algorithm 1, sample from the marginalized distribution via MC (Eq. 36).

The accuracies of both the point predictions and the predictions of the pushforward measure are assessed by comparison with the true values obtained with the simulator (on the test inputs $\{\varvec{\xi }_q^*\}_{q=1}^Q$). We run the solver for each test input to generate the true response, denoted $\tilde{\mathbf {y}}^{*}_q$. For the UQ comparison we again approximate the pdf using KDE (or simply extract the moments) based on $\{\tilde{\mathbf {y}}^{*}_q\}_{q=1}^Q$. The latter approximation is guaranteed to converge to the truth as the number of test inputs increases.

### Darcy Flow: Non-point Source Pollution

The first example is a linear model of steady-state groundwater flow in 2-d. The approach was developed by Kourakos et al. (2012) and implemented in the mSim package.^3^ The model comprises Darcy’s law and a contaminant mass balance in a 2-d polygonal domain $\Omega$ of total area 18.652 km$^2$ containing wells and a stream, and subdivided into polygonal regions of different land use (Fig. 5). Full details of the model and the numerical method can be found in Kourakos et al. (2012). Below we provide a brief description. The model equations are given by:

37 $$\begin{aligned} \begin{aligned} \nabla \cdot \left( K\nabla h\right)&=Q\\ \displaystyle R\frac{\partial C}{\partial t}&=\nabla \cdot \left( \mathbf{D}\nabla C\right) -\nabla \cdot \left( \mathbf{v}C\right) =G \end{aligned} \end{aligned}$$

where $K(\mathbf {x})$ is the hydraulic conductivity, $h(\mathbf {x})$ is the pressure head, $C(\mathbf {x},t)$ is the contaminant concentration, *R* is the retardation factor, $\mathbf{D}$ is the dispersion tensor, $\mathbf{v}$ is the fluid velocity, and *Q* and *G* represent sources/sinks. The contaminant transport equation is replaced by a 1-d approximation and is solved through an ensemble of one-dimensional streamline-based solutions (Kourakos et al. 2012).

The contaminant balance and flow (Darcy) equations are decoupled. The latter is solved using the finite element method based on triangular elements and first-order (linear) shape functions. The boundary conditions are given by: (i) a constant head equal to 30 m on the left boundary; (ii) a general head boundary equal to 40 m with conductance equal 160 m$^3$ day$^{-1}$ on the right boundary; and (iii) no flow on the top and bottom boundaries. Each land use polygon is assigned its own recharge rate. Stream rates are assigned directly to nodes. (Any node closer than 10 m to the stream is considered to be part of the stream.)

We assume that $K(\mathbf {x})$ is log-normally distributed and treat it as an input. The output field upon which we focus is the pressure head, that is, $u(\mathbf {x};K)=h(\mathbf {x})$ in the notation of Sect. 2. We use the input model described in Sect. 2, defining a discretized random field corresponding to realizations of $K(\mathbf {x})=\exp (Z(\mathbf {x}))$ at the nodes $\{\mathbf {x}_k\}_{k=1}^{{k_{y}} }\subset \mathcal{R}$ on the finite element mesh. The covariance function for the random field $Z(\mathbf {x})$ is given by:

38 $$\begin{aligned} c_Z(\mathbf {x},\mathbf {x}') = \sigma _Z^2\exp \left\{ -\frac{(x_1-x_1')^2}{l_1^2}-\frac{(x_2-x_2')^2}{l_2^2}\right\} ,\quad \mathbf {x}=(x_1,x_2)^T\in \mathcal{R}, \end{aligned}$$

where $l_1$ and $l_2$ are correlation lengths. This separable form was suggested in Zhang and Lu (2004) and is used extensively in the literature to model hydraulic permeability fields (often by setting the correlation lengths equal). The generalized variance (value of ${k_{\xi }}$) was chosen to satisfy $\sum _{j=1}^{{k_{\xi }} } \sqrt{\lambda _j}/ \sum _{j=1}^{{k_{y}} } \sqrt{\lambda _j} > 0.98$.

Both the training and test input samples were drawn independently: $$\varvec{\xi }_n \sim \mathcal {N}\left( \mathbf{0},\mathbf{I}\right) $$, $$n=1,\ldots , N$$ to yield $$\{\mathbf {y}_n\}_{n=1}^N$$ for training; and $$\varvec{\xi }_q \sim \mathcal {N}\left( \mathbf{0},\mathbf{I}\right) $$, $$q=1,\ldots , Q$$ to yield $$\{\widetilde{\mathbf {y}}^{*}_q\}_{q=1}^Q$$ for testing and the forward problem (UQ). We set $$Q=5000$$ and $$N\in \{25,50,75,100\}$$. Running the solver with an input generated using the KL truncation necessarily leads to a response surface with intrinsic dimension at most $${k_{\xi }} $$, which was therefore the value chosen for the approximating manifold dimension $${k_{z}} $$. In all of the results presented below, $${k_{y}} = 1933$$ nodes (elements) were used in the simulation. The number of neighbours *P* in the LTSA algorithm was chosen according to the error between the solver response and the predictive mean at the test points. We define a scaled measure of error on each test point as follows:

39 $$\begin{aligned} e_q=||\widetilde{\mathbf {y}}^{*}_q-\overline{\mathbf {y}}_q^*||/||\widetilde{\mathbf {y}}^{*}_q||, \quad q=1,\ldots ,Q, \end{aligned}$$

where $\widetilde{\mathbf {y}}^{*}_q$ is the response predicted by the solver, and $\overline{\mathbf {y}}_q^*$ is the point recovered by application of the pre-image map (23) on the GP *predictive mean* (26). The scaling ensures that the errors are comparable and can be interpreted as percentage errors.

We present results for three stochastic input models:

**M1**: We set $m_Z=\ln (40)$ and $\sigma _Z^2 = 0.2$, yielding^4^ a mean for $k(\mathbf {x})$ of 44.2 m day$^{-1}$, which is close to the default value in the mSim package, and a standard deviation of 13.63 m day$^{-1}$. The correlation lengths were chosen as $l_1=2000$ m and $l_2=1000$ m, which correspond to dimensionless values of 1 / 3 and 2 / 7, respectively. These choices require ${k_{\xi }} =5$ input dimensions to capture $98\%$ of the generalized variance.
**M2**: We set $m_Z=\ln (36.18)$ and $\sigma _Z^2 = 0.4$, again yielding a mean 44.2 m day$^{-1}$ and a standard deviation of 18.80 m day$^{-1}$. We set $l_1=2000$ m and $l_2=1000$ m. ${k_{\xi }} =5$ captures $98\%$ of the generalized variance.
**M3**: We set $m_Z=\ln (40)$, and $\sigma _Z^2 = 0.4$ and reduce the correlation lengths to $l_1=1000$ m and $l_2=500$ m (dimensionless values of 1 / 6 and 1 / 7, respectively). We now require ${k_{\xi }} =15$ to capture $98\%$ of the generalized variance.

**Fig. 1:**
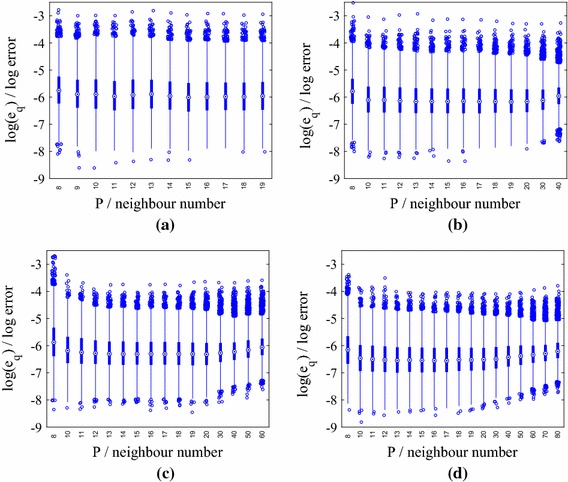
Log normalized error $\ln (e_q)$ in the normalized pressure head prediction for an emulator trained on $N=25$, 50, 75 and 100 points $\mathbf {y}_n$ and tested with $Q=5000$ test points $\widetilde{\mathbf {y}}^{*}_q$ for different nearest neighbour numbers *P* (model **M1**). Predictions were obtained by averaging over hyperparameter and precision posterior samples. **a** 25 training points. **b** 50 training points. **c** 75 training points. **d** 100 training points

**Fig. 2:**
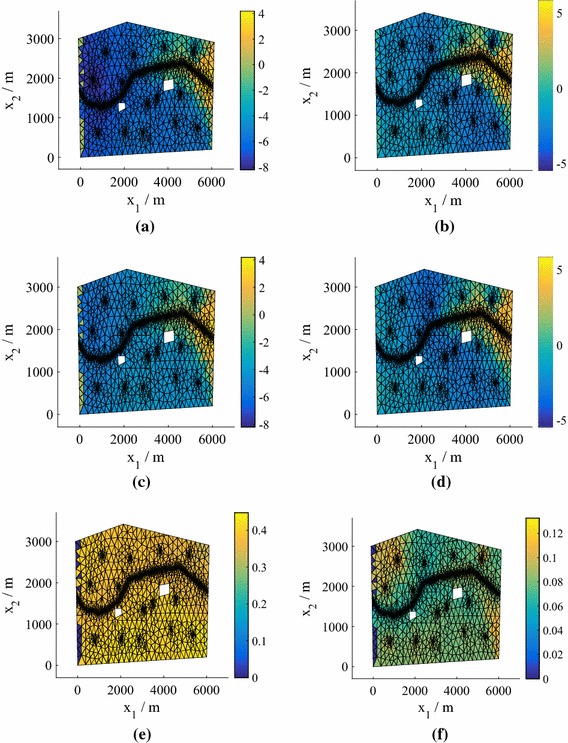
The test predictive mean and standard deviation of the normalized pressure head for the point with highest error from an emulator using $P=15$, corresponding to the relevant boxplot in Fig. 1, for both 25 and 75 training points (model **M1**). **a** True value, $N=25$. **b** True value, $N=75$. **c** Mean of the mean, $N=25$. **d** Mean of the mean, $N=75$. **e** Mean of the standard deviation, $N=25$. **f** Mean of the standard deviation, $N=75$

For model **M1**, the distributions of $\{e_q\}_{n=1}^{Q}$ for different training set sizes *N* are shown as boxplots for increasing values of *P* in Fig. 1. The performance of the emulator is good even for $N=25$ training points (maximum $e_q$ of approximately $e^{-3}$), although there is a clear decrease in the error when *N* is increased to 100. The relationship between the errors and *P* is more complicated. The errors are high for $P<8$ (not shown in the boxplots) at all values of *N* and decrease as *P* increases. This is due to the linear approximation of points in local tangent spaces via PCA in the LTSA algorithm. As more points are added, the approximation improves. As *P* is increased beyond a certain value, however, the errors increase (this is most clearly visible for $N=100$). The reason for this behaviour is that for large enough neighbourhood sizes the linear approximation breaks down. Thus, there is an optimal choice of *P* for each value of *N* and the higher the value of *N* the more sensitive are the errors to the value of *P*. In the subsequent results we use $P=15$ unless otherwise specified.

**Fig. 3:**
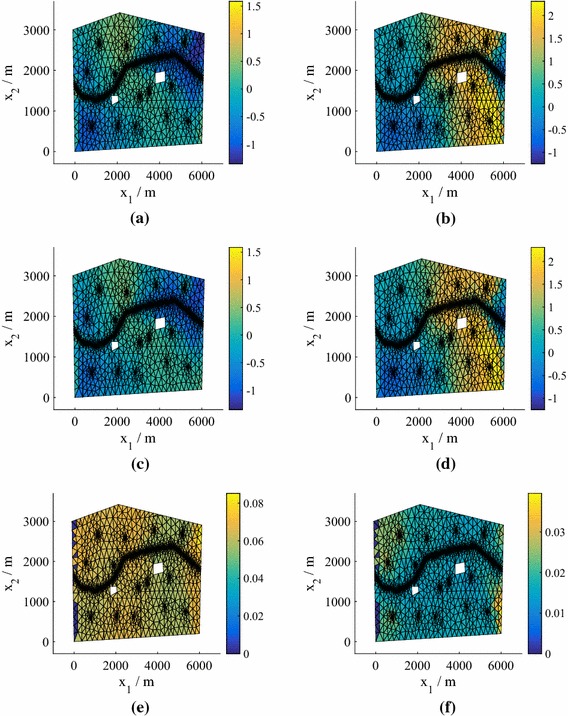
The test predictive mean and standard deviation of the normalized pressure head for a test point with an error near the median of the $P=15$ boxplot in Fig. 1 from emulators using $P=15$, for both 25 and 75 training points (model **M1**). **a** True value, $N=25$. **b** True value, $N=75$. **c** Mean of the mean, $N=25$. **d** Mean of the mean, $N=75$. **e** Mean of the standard deviation, $N=25$. **f** Mean of the standard deviation, $N=75$

In Fig. 2 we plot the *normalized* pressure head prediction (for each coordinate of the predicted pressure head we subtract the mean and divide by the standard deviation) corresponding to the highest $$e_q$$ for both $$N=25 $$ and $$N=50$$ (using $$P=15$$). The normalization highlights the differences between the true values and the predictions (the errors) more clearly. The predicted means of the means (middle row) are the mean predictions averaged over all hyperparameter and precision samples. Also shown (bottom row) are the standard deviations of the predictions averaged over all hyperparameter and precision samples. We observe that the prediction at $$N=75$$ is highly accurate, while the prediction at $$N=25$$ is still reasonably accurate even in this worst case (an outlier in Fig. 1). For both values of *N*, the true values lie within the credible regions. In Fig. 3 we show the corresponding predictions for cases where the errors are close to the medians. Both predictions are highly accurate and again the true values lie inside the credible regions.

**Fig. 4:**
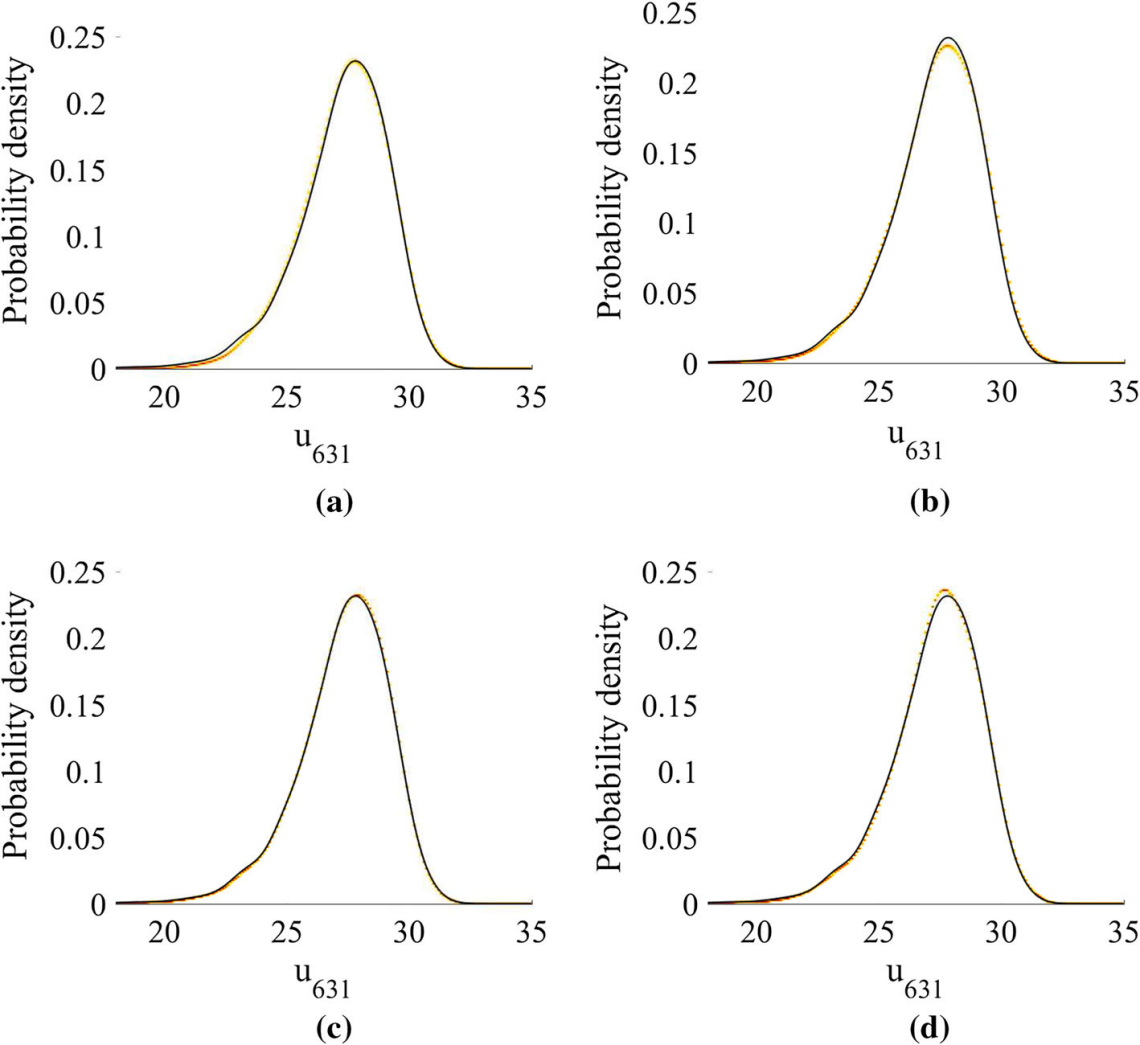
The pdfs of the pressure head response at the spatial coordinate $\mathbf {x}=\mathbf {x}_{631}=\left( 2511,486\right) \in \mathcal{R}$ on the finite difference grid, obtained using kernel density estimation on $Q=5000$ points (Model **M1**). The black line gives the MC prediction using the simulator. The contours show how the emulator predictions vary with hyperparameter, precision and predictive distribution samples. **a** 25 training points, 15 k-NN. **b** 50 training points, 15 k-NN. **c** 75 training points, 15 k-NN. **d** 100 training points, 15 k-NN

We now focus on the forward problem, in which we estimate the marginalized predictive distribution (32) using Algorithm 1. KDE is used to obtain estimates of the pdf of a feature for different predictive posterior, hyperparameter and precision samples, as previously described. The feature we choose is the pressure head at the spatial location $\mathbf {x}= \left( 2511,486\right) \in \mathcal{R}$. We plot a heat map of the pdfs in Fig. 4 for different *N*.

The distributions are accurately estimated for all values of *N*. While the predictions improve as the number of training samples *N* increases, the true value does not always lie within the contours. This is because: (i) as stated earlier, an increased GP predictive variance acts to smooth the density, rather than increase the width of the contours; (ii) by choosing a priori the number of neighbours we also a priori assume a *global* smoothness of the emulator; and (iii) we have a pre-image map $\widehat{\mathbf {f}} : \mathcal {F}\rightarrow \mathcal Y$ for which the error is unknown (as with all methods), but not estimated (as with probabilistic methods).

**Fig. 5:**
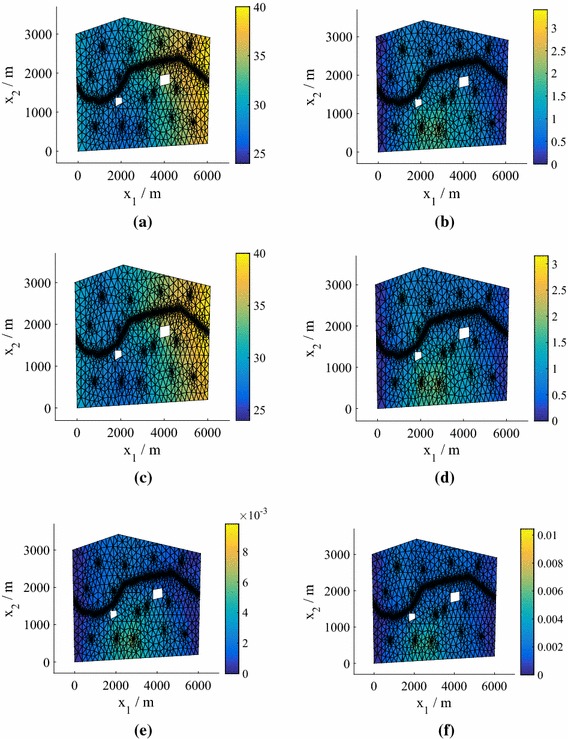
Moments of the mean and standard deviation of the pressure head in Model **M1**. The emulator variation is a consequence of the hyperparameter, precision and predictive distribution samples. We have a single, parameterized realization of the manifold. **a** Monte Carlo mean. **b** Monte Carlo standard deviation. **c** Mean of the mean for 25 training points. **d** Mean of the standard deviation for 25 training points. **e** Standard deviation of the mean for 25 training points. **f** Standard deviation of the standard deviation for 25 training points

We can find the means and standard deviations across the samples obtained for different predictive posterior, hyperparameter and precision samples using Algorithm 1. We obtain distributions over the moments of the marginalized predictive distribution (32). In Fig. 5 we plot the mean and standard deviation of the marginalized predictive mean and standard deviation for $N=25$, with comparisons to the true values obtained by finding the mean and standard deviation across the test responses $\{\widetilde{\mathbf {y}}^{*}_q\}_{q=1}^Q$. Even for this low number of training points the results are highly accurate.

**Fig. 6:**
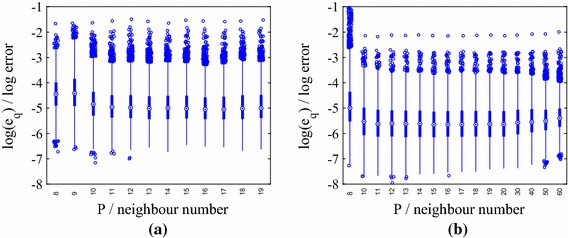
Log normalized error $\ln (e_q)$ in the normalized pressure head prediction for an emulator trained on $N=25$ and 75 points $\mathbf {y}_n$ and tested with $Q=5000$ test points $\widetilde{\mathbf {y}}^{*}_q$ for different nearest neighbour numbers *P* (model **M2**). Predictions were obtained by averaging over hyperparameter and precision posterior samples. **a** 25 training points. **b** 75 training points

We now consider Model **M2**, in which we increase the variance of the stochastic input, while keeping the mean fixed. For this example we again set $l_1 = 2000$ m and $l_2 = 1000$ m, requiring ${k_{\xi }} =5$. The distributions of $\{e_q\}_{q=1}^{Q}$ for different training set sizes *N* and increasing *P* are shown in Fig. 6. We observe trends similar to those observed using Model **M1**, although the increased variance leads to larger errors at fixed *N* and *P* (higher maxima and minima). With the exception of an isolated outlier (shown later), the predictions are nevertheless accurate for $N=75$.

**Fig. 7:**
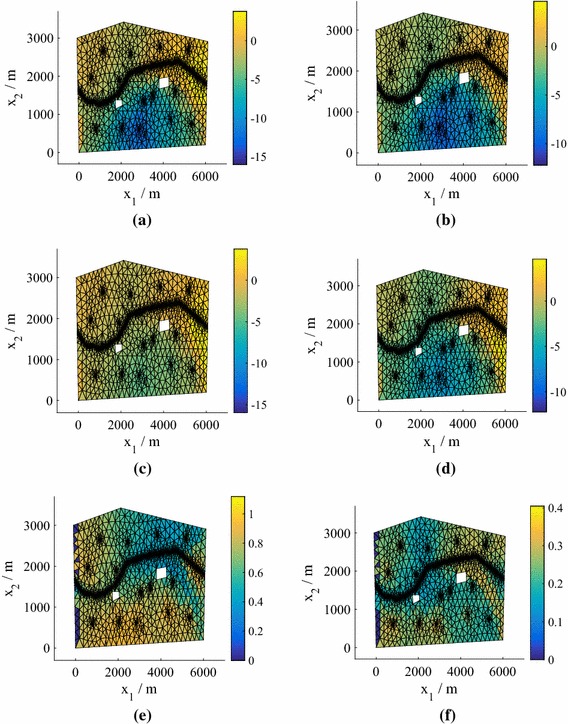
The test predictive mean and standard deviation of the normalized pressure head in the case of the highest errors $e_q$ in Fig. 6 for $P=15$ and $N=25$ and 75 training points (Model **M2**). **a** True value, $N=25$. **b** True value, $N=75$. **c** Mean of the mean, $N=25$. **d** Mean of the mean, $N=75$. **e** Mean of the standard deviation, $N=25$. **f** Mean of the standard deviation, $N=75$

**Fig. 8:**
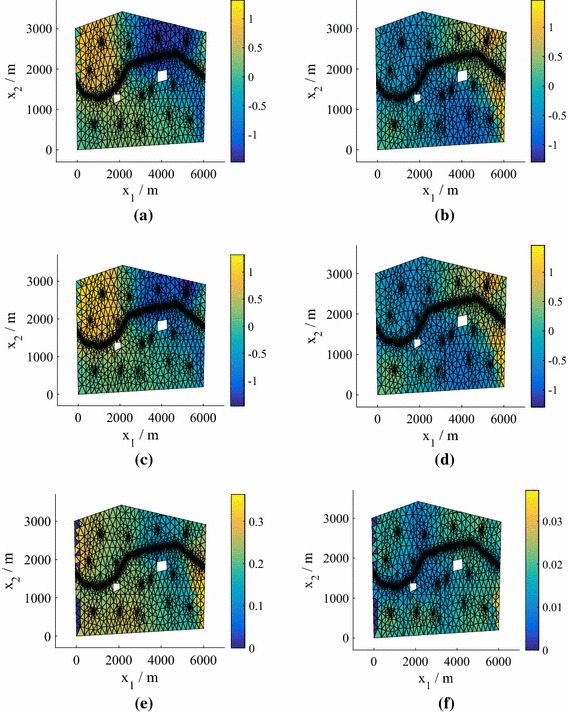
The test predictive mean and standard deviation of the normalized pressure head in the case of errors $e_q$ near the median in Fig. 6 for $P=15$ and $N=25$ and 75 training points (Model **M2**). **a** True value, $N=25$. **b** True value, $N=75$. **c** Mean of the mean, $N=25$. **d** Mean of the mean, $N=75$. **e** Mean of the standard deviation, $N=25$. **f** Mean of the standard deviation, $N=75$

The worst case (highest $e_q$) for $P=15$ is shown in Fig. 7 for $N=25$ and 75 points (see Fig. 6). As before the top row is the test (solver prediction), while the middle and bottom rows are the mean prediction and standard deviation of the prediction averaged over all hyperparameter and precision samples. The true values lie within the credible regions, although for this model a higher number of training points are required to ensure that even the worst-case predictions are accurate. Figure 8 demonstrates the quality of the predicted responses when the errors are at the median in the $P=15$ boxplots in Fig. 6. Here, even $N=25$ provides accurate results.

**Fig. 9:**
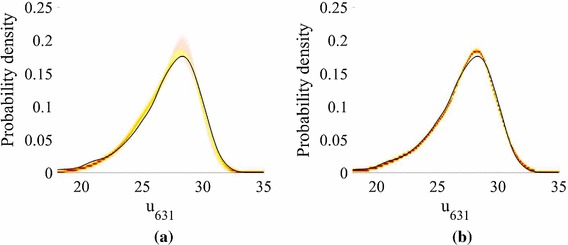
The pdfs of the pressure head response at the spatial coordinate $\mathbf {x}=\mathbf {x}_{631}=\left( 2511,486\right)$ on the finite difference grid, obtained using kernel density estimation on $Q=5000$ points (Model **M2**). The black line gives the MC prediction using the simulator. The contours show how the emulator predictions vary with hyperparameter, precision and predictive distribution samples. **a** 25 training points, 15 k-NN. **b** 75 training points, 15 k-NN

**Fig. 10:**
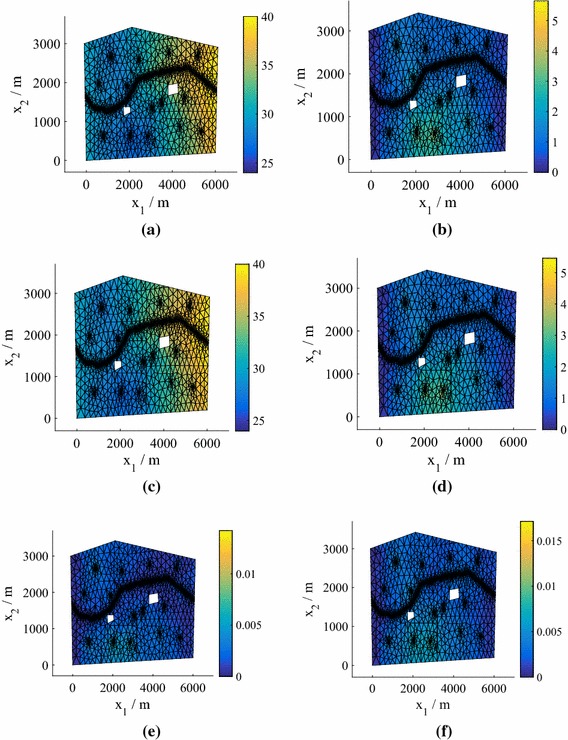
Moments of the mean and standard deviation of the pressure head for $P=15$, $N=75$ (Model **M2**). The emulator variation is a consequence of the hyperparameter and predictive distribution samples. We have a single, parameterized realization of the manifold. **a** Monte Carlo mean. **b** Monte Carlo standard deviation. **c** Mean of the mean for 75 training points. **d** Mean of the standard deviation for 75 training points. **e** Standard deviation of the mean for 75 training points. **f** Standard deviation of the standard deviation for 75 training points

Figure 9 shows heat maps of the pdfs of the pressure head at the spatial location $\mathbf {x}= \left( 2511,486\right)$ for different *N* (generated using KDE) in the case of Model **M2**. Using $N=75$ we achieve very good agreement with the MC prediction based on the simulator results (test points), although again the true value does not lie within the contours. For $N=25$, we plot the mean and standard deviation of the marginalized predictive mean and standard deviation in Fig. 10, with a comparison to the true values obtained from $\{\widetilde{\mathbf {y}}^{*}_q\}_{q=1}^Q$. The predictions are highly accurate. In fact, even for $N=25$ (not shown to conserve space) the mean was very accurate and the standard deviation exhibited only slight differences from the true value.

**Fig. 11:**
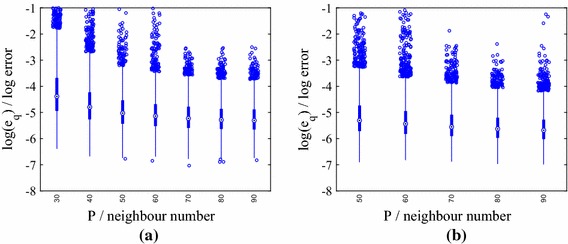
Log normalized error between true and predictive mean in the normalized pressure head prediction from an emulator trained on 300 and 500 points $\mathbf {y}_n$ and interrogated with $Q=5000$ test points $\tilde{\mathbf {y}}^{*}_q$ for different nearest neighbour numbers *P* (Model **M3**). Predictions were obtained by averaging over hyperparameter and precision posterior samples. **a** 300 training points. **b** 500 training points

**Fig. 12:**
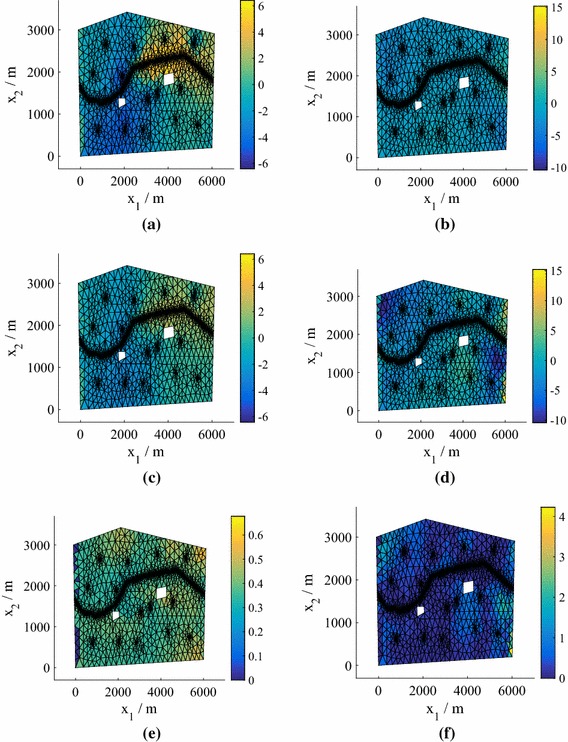
The test predictive means and standard deviations for predictions of the normalized pressure head with the highest errors from emulators using $P=70$, $N=300$ and with $P=80$, $N=500$, corresponding to the relevant boxplots in Fig. 11 (Model **M3**). **a** True value, $N=300$. **b** True value, $N = 500$. **c** Mean of the mean, $N=300$. **d** Mean of the mean, $N=500$. **e** Mean of the standard deviation, $N=300$. **f** Mean of the standard deviation, $N=500$

**Fig. 13:**
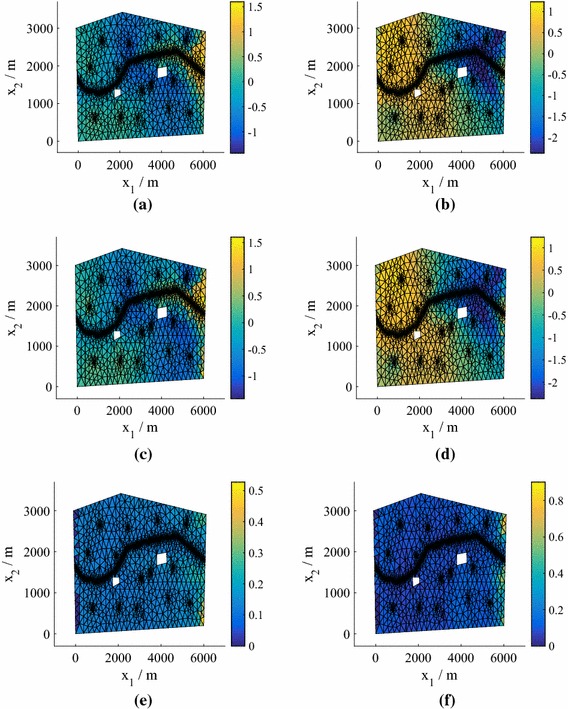
The test predictive means and standard deviations for predictions of the normalized pressure head with errors at the median from emulators using $P=70$, $N=300$ and with $P=80$, $N=500$, corresponding to the relevant boxplots in Fig. 11 (Model **M3**). **a** True value, $N=300$. **b** True value, $N = 500$. **c** Mean of the mean, $N=300$. **d** Mean of the mean, $N=500$. **e** Mean of the standard deviation, $N=300$. **f** Mean of the standard deviation, $N=500$

For Model **M3** (decreased correlation lengths, high standard deviation and ${k_{\xi }} =15$), the distributions of $\{e_q\}_{q=1}^{Q}$ for increasing *N* and *P* in are shown in Fig. 11. In this case it is clear that a much higher value of *P* ($P>60$, giving a similar neighbourhood radius in-line with the increased sample density) is required to obtain a reasonable accuracy. For $N=500$ and $P=80$, there are a small (9 out of 5000) number of outliers with low accuracy, while the errors for the remaining points satisfy $\ln (e_q)<-3.25$. The worst cases (highest $e_q$) for $P=70$, $N=300$ and $P=80$, $N=500$ are shown in Fig. 12, and in Fig.13 we show predicted responses with errors at the medians for the same values of *P* and *N*. There are noticeable differences in the worst cases, although the qualitative agreement is very good at both values of *N*. For the median error cases both emulators perform extremely well.

**Fig. 14:**
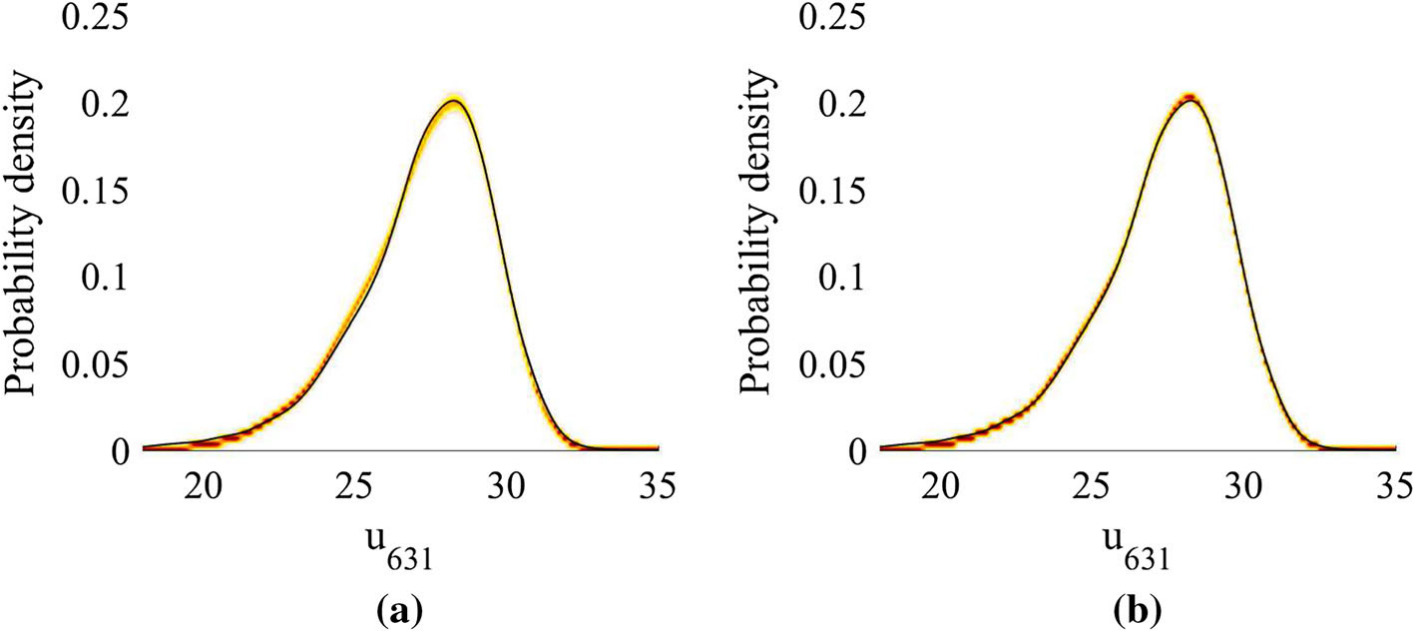
The pdfs of the pressure head response at the spatial coordinate $\mathbf {x}=\mathbf {x}_{631}=\left( 2511,486\right)$ on the finite difference grid, obtained using kernel density estimation on $Q=5000$ points (Model **M3**). The black line gives the MC prediction using the simulator. The contour shows how the emulator predictions vary with hyperparameter, precision and predictive distribution samples. **a** 300 training points, $P=70$. **b** 500 training points, $P=80$

**Fig. 15:**
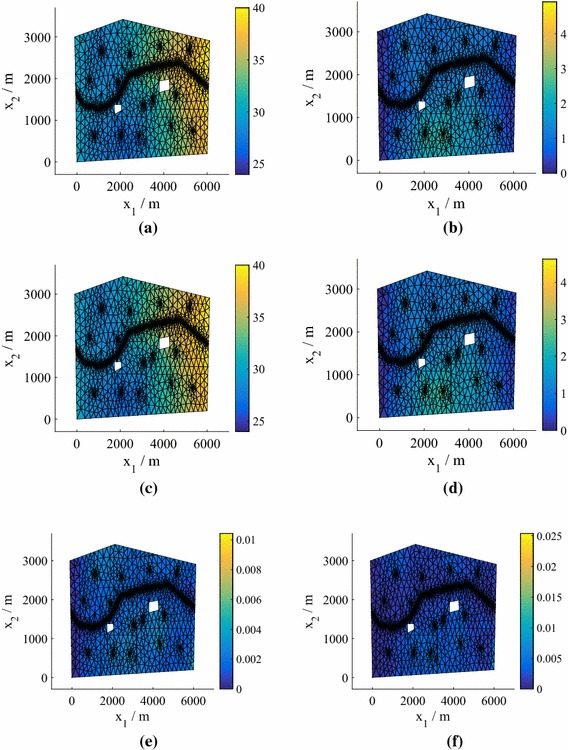
Moments of the mean and standard deviation for $P=80$, $N=500$ of the pressure head (Model **M3**). The emulator variation is a consequence of the hyperparameter and predictive distribution samples. We have a single, parameterized realization of the manifold. **a** Monte Carlo mean. **b** Monte Carlo standard deviation. **c** Mean of the mean for 500 training points. **d** Mean of the standard deviation for 500 training points. **e** Standard deviation of the mean for 500 training points. **f** Standard deviation of the standard deviation for 500 training points

In Fig. 14 we show the heat maps of the pdfs of the pressure head at $\mathbf {x}= \left( 2511,486\right)$ for different *N*. For both values of *N* there is very good agreement with the simulator result and the true value this time lies within the contours. For $N=500$, we show the mean and standard deviation of the marginalized predictive mean and standard deviation in Fig. 15, with a comparison to the true values obtained from $\{\widetilde{\mathbf {y}}^{*}_q\}_{q=1}^Q$. The predictions are again highly accurate (which was also the case for $N=300$).

### Richards Equation: Unsaturated Flow in Porous Media

Consider a single-phase flow through a 3-d porous region $\mathcal{R}\subset \mathbb {R}^3$ containing unsaturated soil with a random permeability field. The vertical flow problem can be solved using Richards equation (Darcy’s law combined with a mass balance). There are three standard forms of Richards equation: the pressure head based (*h*-based) form; the water content-based ($\theta$-based) form; and the mixed-based form. For flow in saturated or layered soils, the *h*-based form is particularly appropriate (Huang et al. 1996; Shahraiyni and Ataie-Ashtiani 2011).

The *h*-based form with an implicit or explicit finite difference (FD) scheme has been shown to provide good accuracy, although this approach may result in high mass balance errors (Zarba et al. 1990; Huang et al. 1996). The mixed-based form, on the other hand, exhibits low mass balance errors with highly accurate predictions using a fully implicit FD scheme (Ray and Mohanty 1992; Zarba et al. 1990; Celia et al. 1987). The latest work of Shahraiyni and Ataie-Ashtiani (2011) showed that a fully implicit FD scheme with a standard chord slope (CSC) approximation (Rathfelder and Abriola 1994) not only solved the mass balance problem of the *h*-based form but also improved convergence. Thus, in the paper we adopt this approach, although other numerical formulations are by no means precluded. The *h*-based form of Richards equation can be written as follows:

40 $$\begin{aligned} u(h) \frac{\partial h}{\partial t} - \nabla \cdot \textit{K}(h) \nabla (h+x_3)=0, \qquad (\mathbf x ,t) \in \mathcal{R} \times (0, T], \end{aligned}$$

where *h* is the pressure head, $u(h)=\partial \theta / \partial h$ is the specific moisture capacity, in which $\theta$ is the moisture content, *K*(*h*) is the unsaturated hydraulic conductivity, and ${{\varvec{x}}}=(x_1,x_2,x_3)^T$ is the spatial coordinate, in which $x_3$ is the vertical coordinate. The nonlinear functions $\theta (h)$ and *k*(*h*) can take on different forms. For example, in Haverkamp et al. (1977), a least square fit to experimental data was used to derive:

41 $$\begin{aligned} \begin{aligned} \displaystyle \theta (h)&= \frac{\alpha _1 (\theta _s-\theta _r)}{\alpha _1 + |h|^{\alpha _2}} +\theta _r, \\ \displaystyle \textit{K}(h)&=K_s (\mathbf {x}) \frac{\alpha _3}{\alpha _3 +|h|^{\alpha _4}}, \end{aligned} \end{aligned}$$

where $\theta _r$ and $\theta _s$ are the residual the saturated water contents, $K_s (\mathbf{x})$ is the saturated hydraulic conductivity, and $\alpha _1$, $\alpha _2$, $\alpha _3$ and $\alpha _4$ are fitting parameters. We adopt the relationships (41) and use the parameter values in Haverkamp et al. (1977): $\alpha _1=1.611\times 10^6$, $\alpha _2=3.96$, $\alpha _3=1.175\times 10^6$, $\alpha _4=4.74$, $\theta _s=0.287$ and $\theta _r=0.075$. The domain $\mathcal{R}$ is taken to be $20 \text{ cm } \times 20 \text{ cm }\times 20 \text{ cm }$. $K_s({\mathbf {x}})$ is treated as a random field input with a log-normal distribution ($K_s({\mathbf {x}})=\exp (Z(\mathbf {x})$), again discretized using the Karhunen–Loève theorem. We generate realizations of a corresponding discrete random field on an $n_1\times n_2 \times n_3$ finite difference grid (${k_{y}} =n_1n_2n_3$), with grid spacings $\Delta x_1$, $\Delta x_2$ and $\Delta x_3$ in the directions $x_1$, $x_2$ and $x_3$, respectively. The output field of interest is again the pressure head, at a fixed time *T*. Thus, we set $u(\mathbf {x};K)=h(\mathbf {x},T)$.

The boundary conditions are those used in Haverkamp et al. (1977), corresponding to laboratory experiments of infiltration in a plexiglass column packed with sand. Along the top boundary (surface) $x_3=20$ cm, the pressure head is maintained at $h=-20.7$ cm ($\theta =0.267$ cm$^3$ cm$^{-3}$), and along the bottom boundary $x_3=0$ cm, it is maintained at $h=-\,61.5$ cm. At all other boundaries a no-flow condition is imposed: $\nabla h \cdot \mathbf{n}=0$, where $\mathbf{n}$ is the unit, outwardly pointing normal to the surface. The initial condition is $h(\mathbf {x},0)=-\,61.5$ cm.

The covariance function for the random field $Z(\mathbf {x})$ is again of the form:

42 $$\begin{aligned} c_Z(\mathbf {x},\mathbf {x}') = \sigma _Z^2\exp \left\{ -\frac{(x_1-x_1')^2}{l_1^2}-\frac{(x_2-x_2')^2}{l_2^2}-\frac{(x_3-x_3')^2}{l_3^2}\right\} , \end{aligned}$$

where the $l_i$ are correlation lengths, chosen as $l_1=l_2=l_3=7.5$ cm. The mean $m_Z$ and variance $\sigma _Z^2$ are chosen such that the mean and standard deviation of $K(\mathbf {x})$ are 0.0094 cm s$^{-1}$ (Shahraiyni and Ataie-Ashtiani 2011; Haverkamp et al. 1977) and 0.00235 cm s$^{-1}$ (25 % of the mean), respectively. The generalized variance satisfies $\sum _{i=1}^{{k_{\xi }} } \sqrt{\lambda _i}/ \sum _{i=1}^n \sqrt{\lambda _i} = 0.75$ for ${k_{\xi }} =15$.

The training and test input samples were drawn independently: $\varvec{\xi }_n \sim \mathcal {N}\left( \mathbf{0},\mathbf{I}\right)$ and $\varvec{\xi }_q \sim \mathcal {N}\left( \mathbf{0},\mathbf{I}\right)$ to yield $\{\mathbf {y}_n\}_{n=1}^N$ for training and $\{\widetilde{\mathbf {y}}^{*}_q\}_{q=1}^Q$ for testing and UQ. We set $Q=5000$ and $N\le 800$. As before, the manifold dimension was set to ${k_{z}} ={k_{\xi }}$. The number of neighbours *P* and the number of training points *N* were chosen as in the first example by examining the errors $e_q=||\widetilde{\mathbf {y}}^{*}_q-\overline{\mathbf {y}}_q^*||/||\widetilde{\mathbf {y}}^{*}_q||$ on the test set, where again $\widetilde{\mathbf {y}}^{*}_q$ is the solver output (truth) and $\overline{\mathbf {y}}_q^*$ is emulator prediction based on the GP predictive mean (26).

Equation (40) was solved using a finite difference scheme with first-order differencing for the first-order derivatives, central differencing for the second-order derivatives and a fully implicit backward Euler time stepping scheme. A picard iteration scheme is used (Celia et al. 1990) at each time step. Details are provided in “Appendix C”.

**Fig. 16:**
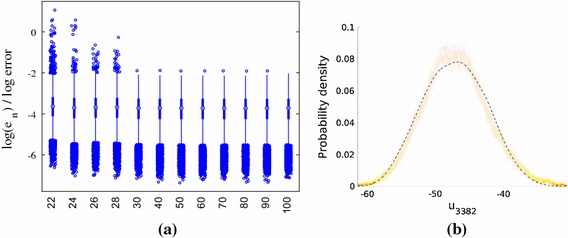
**a** Log normalized error $\ln (e_q)$ for an emulator trained on $N=800$ points $\mathbf {y}_n$ and tested with $Q=5000$ test points $\widetilde{\mathbf {y}}^{*}_q$ for different nearest neighbour numbers *P*. Predictions were obtained by averaging over hyperparameter and precision posterior samples. **b** The pdfs of the pressure head response at the location $\mathbf {x}=(10.4,10.4,10.4)^T$ ($N=800$), obtained using kernel density estimation on $\{\widetilde{\mathbf {y}}^{*}_q\}_{q=1}^Q$. The black line gives the MC prediction using the simulator. **a** 800 training points. **b** 800 training points, 30 k-NN

We followed the procedure of the first example. Training point numbers below 600 led to inaccurate results. For $N=600$, the results were reasonably accurate but to achieve good accuracy we required $N>700$. We present the results for $N=800$. The pressure head is normalized as in the first example in order to highlight the errors in the predictions more clearly. In Fig. 16a we plot the log normalized error $\ln (e_q)$ for an emulator trained on $N=800$ points $\mathbf {y}_n$ and tested with $Q=5000$ points $\widetilde{\mathbf {y}}^{*}_q$ for different nearest neighbour numbers $P> 20$ (averaging over hyperparameter and precision posterior samples). For $P\le 20$ the errors were high, with the same trend as seen in the first example.

**Fig. 17:**
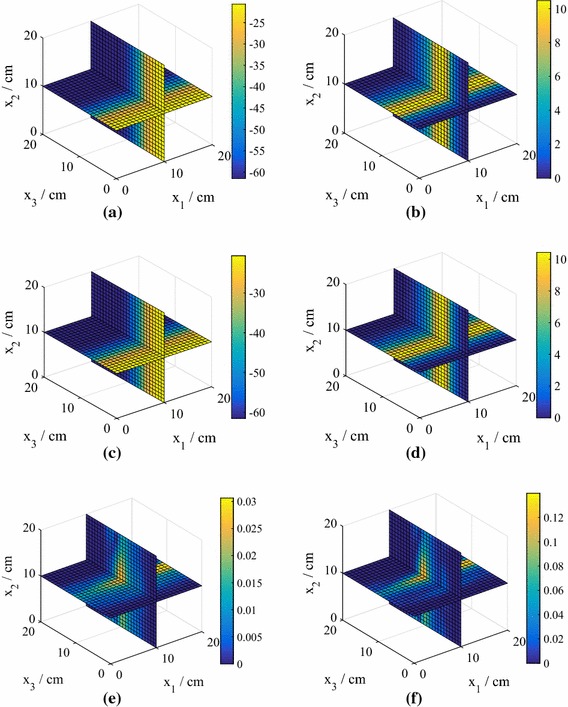
Moments of the mean and standard deviation of the pressure head for $P=30$, $N=800$. Shown are the planes $x_1=10.4$ cm and $x_2=10.4$ cm. The emulator variation is a consequence of the hyperparameter and predictive distribution samples. We have a single, parameterized realization of the manifold. **a** Monte Carlo mean. **b** Monte Carlo standard deviation. **c** Mean of the mean. **d** Mean of the standard deviation. **e** Standard deviation of the mean. **f** Standard deviation of the standard deviation

We use Algorithm 1 and KDE to obtain predictions of the pdf of a feature of the response. We choose as a feature the pressure head at the location $\mathbf {x}=(10.4,10.4,10.4)^T$ (grid point number 4411). The distributions are shown in Fig. 16b for $N=800$. We can again find the means and standard deviations across predictive posterior, hyperparameter and precision samples to obtain distributions over the moments of the marginalized distribution (32). These are plotted in Fig. 17, alongside comparisons to the true values obtained from $\{\widetilde{\mathbf {y}}^{*}_q\}_{q=1}^Q$. These results show that the emulator performs extremely well, accurately capturing both the mean and standard deviation with high precision.

## Numerical Computation

LTSA naturally lends itself to parallelization since almost all computations are performed on each neighbourhood independently. After merging threads we need only solve an eigenvalue problem for an $N\times N$ matrix. Similarly, independent Gaussian processes across latent dimensions leads to a natural parallelization framework.

For large sample sizes and feature space dimensions saving each $Q_i$ can become infeasible ($N \times {k_{y}} \times {k_{z}}$ elements). Similarly, for large sample and neighbourhood sizes saving *f* can become infeasible ($N\times k^2$ elements). In such cases, these tensors may be saved to file or re-calculated online.

The scalability of our approach is limited by the computational complexity of Gaussian processes $\mathcal {O}\left( N^3\right)$. However, this can be alleviated by using sparse Gaussian process regression models. These models introduce *m* inducing points, reducing computational complexity to $\mathcal {O}\left( m^2 N\right)$. We may also use active learning to reduce the number of samples required.

## Summary and Conclusions

In this paper we developed a new approach to the emulation of a model involving a random field input and a field output, with a focus on problems arising in groundwater flow modelling. The main challenges are the high input and output space dimensionalities, which we dealt with using a KL expansion and manifold learning, respectively. We implemented LTSA on the given outputs (training data), which allowed us to perform Bayesian inference in a low-dimensional feature space. Furthermore, we developed a framework for UQ in such problems by marginalizing over the inputs, either analytically (the mean and possibly in some cases the standard deviation) or using MC sampling.

Testing the emulation method on two examples reveals that it performs well in certain cases. When the variance of the log-normal input is high or the correlation lengths of the normal process $Z(\mathbf {x})$ are short, the accuracy suffers, as is found in all other approaches. Nevertheless, the accuracy in terms of the forward UQ problem is high even in such cases for the examples considered. (Of course, further increases in the variance and correlation lengths would eventually lead to unacceptably poor performance.)

The major drawback of the KL expansion approach (and similarly with circulant embedding) is the curse of dimensionality as the number of retained coefficients grows. Some progress can be made in this regard by using a Smolyak algorithm (Smolyak 1963) for sampling or incremental local tangent space alignment (Liu et al. 2006) combined with active learning (Settles 2012), but the gains will be limited. Our method, in common with other methods except direct Monte Carlo or ROMs, is therefore potentially limited, given current computational resources, to problems in which the domain size is at most a few multiples of the shortest correlation length. The assumption of independence of the feature vector coordinates is also sub-optimal. Since the number of coordinates is small, however, this assumption can easily be relaxed by adopting, e.g. a convolved GP approach.

## Appendix A: Moments of the Marginal Distribution Over $\mathbf {z}$

Focusing on the *i*th feature of $\mathbf {z}$, we find the first two moments, i.e. the mean and variance, of the marginal distribution $p\left( z_i|\mathcal {D},\varvec{\theta }_i,\beta _i\right)$. Following Girard and Murray-Smith (2003), we approximate $p\left( z_i|\mathcal {D},\varvec{\theta }_i,\beta _i\right)$ as a Gaussian with mean *m* and variance *v*:

A1 $$\begin{aligned} p\left( z_i|\mathcal {D},\varvec{\theta }_i,\beta _i\right) = \int p(z_i|\varvec{\xi }',\mathcal {D},\varvec{\theta }_i,\beta _i)p(\varvec{\xi }') \text {d}\varvec{\xi }' \approx \mathcal {N} \left( m,v\right) . \end{aligned}$$

Below we use the notation $\mathbb {E}_\chi [\cdot ]$ and $\mathbb {V}\text{ ar }_\chi (\cdot )$ to denote an expectation and variance operator with respect to a random variable $\chi$, respectively. Using Fubini’s theorem and the laws of total expectation and variance, the moments are then given by:

A2 $$\begin{aligned} \begin{aligned} m&= \int z_i' \,p\left( z_i'|\mathcal {D},\varvec{\theta }_i,\beta _i\right) \text {d}z_i' \\&= \int z_i' \left[ \int p\left( z_i'|\varvec{\xi }',\mathcal {D},\varvec{\theta }_i,\beta _i\right) p(\varvec{\xi }') d\varvec{\xi }' \right] \text {d}z_i' \\&= \int \left[ \int z_i'\,p\left( z_i'|\varvec{\xi }',\mathcal {D},\varvec{\theta }_i,\beta _i\right) dz_i'\right] p(\varvec{\xi }') \text {d}\varvec{\xi }' \\&= \mathbb {E}_{\varvec{\xi }} \left[ \mathbb {E}_{z_i}\left[ z_i|\varvec{\xi },\mathcal {D},\varvec{\theta }_i,\beta _i\right] \right] \\&= \mathbb {E}_{\varvec{\xi }} \left[ \mu (\varvec{\xi })\right] \\&= \mathbb {E}_{\varvec{\xi }} \left[ \mathbf {c}_h(\varvec{\xi },\pmb {\Xi };\varvec{\theta }_i)^T \left( \mathbf{C}_i+\beta _i^{-1} \mathbf{I}\right) ^{-1}\mathbf {z}_{:,i}\right] \\&= \mathbb {E}_{\varvec{\xi }}\left[ \mathbf {c}_h(\varvec{\xi },\pmb {\Xi };\varvec{\theta }_i)\right] ^T \left( \mathbf{C}_i+\beta _i^{-1} \mathbf{I}\right) ^{-1}\mathbf {z}_{:,i} \end{aligned} \end{aligned}$$

and:

A3 $$\begin{aligned} \begin{aligned} v&= \int (z_i')^2\,p\left( z_i'|\mathcal {D},\varvec{\theta }_i,\beta _i\right) \text {d}z_i' - m^2 \\&= \int (z_i')^2 \left[ \int p\left( z_i'|\varvec{\xi }',\mathcal {D},\beta _i\right) p(\varvec{\xi }') \text {d}\varvec{\xi }' \right] dz_i' - m^2 \\&= \mathbb {E}_{\varvec{\xi }} \left[ \mathbb {V}\text{ ar }_{z_i}\left( z_i|\varvec{\xi },\mathcal {D},\varvec{\theta }_i,\beta _i\right) \right] + \mathbb {V}\text{ ar }_{\varvec{\xi }}\left( \mathbb {E}_{z_i}\left[ z_i|\varvec{\xi },\mathcal {D},\varvec{\theta }_i,\beta _i\right] \right) \\&= \mathbb {E}_{\varvec{\xi }}\left[ \sigma ^2(\varvec{\xi })\right] + \mathbb {V}\text{ ar }_{\varvec{\xi }}\left( \mu (\varvec{\xi })\right) \\&= \mathbb {E}_{\varvec{\xi }}\left[ \sigma ^2(\varvec{\xi })\right] + \mathbb {E}_{\varvec{\xi }}\left[ \mu (\varvec{\xi })^2\right] - m^2 \\&= \mathbb {E}_{\varvec{\xi }}\left[ c_h(\varvec{\xi },\varvec{\xi };\varvec{\theta }_i) - \mathbf {c}_h(\varvec{\xi },\pmb {\Xi };\varvec{\theta }_i)^T\left( \mathbf {C}+ \beta _i^{-1} \mathbf{I}\right) ^{-1} \mathbf {c}_h(\varvec{\xi },\pmb {\Xi };\varvec{\theta }_i)\right] \\&\quad +\, \mathbb {E}_{\varvec{\xi }}\left[ \left( \mathbf {c}_h(\varvec{\xi },\pmb {\Xi };\varvec{\theta }_i)^T \left( \mathbf{C}_i+\beta _i^{-1} \mathbf{I}\right) ^{-1}\mathbf {z}_{:,i}\right) ^2 \right] - m^2\\&= \mathbb {E}_{\varvec{\xi }}\left[ c_h(\varvec{\xi },\varvec{\xi };\varvec{\theta }_i) \right] - m^2\\&-\left[ \left( \mathbf{C}_i+\beta _i^{-1} \mathbf{I}\right) ^{-1}-\left( \left( \mathbf{C}_i+\beta _i^{-1}\mathbf{I}\right) \mathbf {z}_{:,i}\right) ^2 \right] \mathbb {E}_{\varvec{\xi }}\left[ \mathbf {c}_h(\varvec{\xi },\pmb {\Xi };\varvec{\theta }_i)^T\mathbf {c}_h(\varvec{\xi },\pmb {\Xi };\varvec{\theta }_i) \right] . \end{aligned} \end{aligned}$$

## Appendix B: Kernel Expectation

Given a squared exponential kernel and a Gaussian stochastic input distribution, we are able to analytically find the mean and variance of the marginalized latent predictive distribution. This kernel takes the form:

B1 $$\begin{aligned} c_h(\varvec{\xi },\varvec{\xi }';\varvec{\theta }_i)= s \exp \left( -\frac{1}{2}\left( \varvec{\xi }-\varvec{\xi }'\right) ^T\mathbf {A}\left( \varvec{\xi }-\varvec{\xi }'\right) \right) , \end{aligned}$$

where $\mathbf {A}$ is a diagonal matrix whose elements are inversely proportional to the correlation lengths across input dimensions. For computational convenience, we write this covariance function in Gaussian function form with normalizing constant $a=\left( 2\pi \right) ^{{k_{\xi }} /2}|\mathbf {A}| ^{\frac{1}{2}}s$:

B2 $$\begin{aligned} c_h(\varvec{\xi },\varvec{\xi }';\varvec{\theta }_i) = a\mathcal {N}_{\varvec{\xi }}\left( \varvec{\xi }',\mathbf {A}\right) . \end{aligned}$$

where the notation $\mathcal {N}_{\pmb {\chi }}\left( \cdot ,\cdot \right)$ denotes a normal distribution over a random vector $\pmb {\chi }$, with mean and covariance matrix given by the first and second arguments, respectively. We wish to evaluate:

B3 $$\begin{aligned} \begin{aligned} \mathbb {E}_{\varvec{\xi }}\left[ c_h(\varvec{\xi },\varvec{\xi };\varvec{\theta }_i) \right]&= a, \\ \mathbb {E}_{\varvec{\xi }}\left[ \mathbf {c}_h(\varvec{\xi },\pmb {\Xi };\varvec{\theta }_i)\right]&= \mathbb {E}_{\varvec{\xi }}\left[ \mathbf {c}_h(\varvec{\xi },\varvec{\xi };\varvec{\theta }_i)\right] = a\int \mathcal {N}_{\varvec{\xi }}\left( \varvec{\xi },\mathbf {A}\right) \mathcal {N}_{\varvec{\xi }}\left( \pmb {\mu },\Sigma _{\varvec{\xi }}\right) \text {d}\varvec{\xi }, \\ \mathbb {E}_{\varvec{\xi }}\left[ \mathbf {c}_h(\varvec{\xi },\pmb {\Xi };\varvec{\theta }_i)^T \mathbf {c}_h(\varvec{\xi },\pmb {\Xi };\varvec{\theta }_i)\right]&= \mathbb {E}_{\varvec{\xi }}\left[ \mathbf {c}_h(\varvec{\xi },\varvec{\xi };\varvec{\theta }_i)\mathbf {c}_h(\varvec{\xi },\varvec{\xi };\varvec{\theta }_i)\right] \\&= a^2\int \mathcal {N}_{\varvec{\xi }}\left( \varvec{\xi },\mathbf {A}\right) \mathcal {N}_{\varvec{\xi }}\left( \varvec{\xi },\mathbf {A}\right) \mathcal {N}_{\varvec{\xi }}\left( \pmb {\mu },\Sigma _{\varvec{\xi }}\right) \text {d}\varvec{\xi }, \end{aligned} \end{aligned}$$

where $\left( \pmb {\mu },\Sigma _{\varvec{\xi }}\right)$ are the stochastic input distribution moments. The solutions can be found by using the product of Gaussians rule:

B4 $$\begin{aligned} \begin{aligned} \mathbb {E}_{\varvec{\xi }}\left[ \mathbf {c}_h(\varvec{\xi },\varvec{\xi };\varvec{\theta }_i) \right]&= a \mathcal {N}_{\pmb {\mu }}\left( \varvec{\xi },\mathbf {A}+\Sigma _{\varvec{\xi }}\right) , \\ \mathbb {E}_{\varvec{\xi }}\left[ \mathbf {c}_h(\varvec{\xi },\varvec{\xi };\varvec{\theta }_i)\mathbf {c}_h(\varvec{\xi },\varvec{\xi };\varvec{\theta }_i)\right]&= a^2 \mathcal {N}_{\varvec{\xi }}\left( \varvec{\xi },2\mathbf {A}\right) \mathcal {N}_{\pmb {\mu }} \left( \varvec{\xi },\Sigma _{\varvec{\xi }} + \frac{\mathbf {A}}{2}\right) . \end{aligned} \end{aligned}$$

## Appendix C: Numerical Algorithm for Richards Equation

Let $\psi _{i',j',k'}^{n',m'}$ denote the value of a quantity $\psi$ at time step $n'$ (time $t=n'\Delta t$ for a constant time step $\Delta t$), at Picard iteration $m'$ and at the spatial location $x_1=i'\Delta x_1$, $x_2=j'\Delta x_2$ and $x_3=k'\Delta x_3$. The spatial and temporal discretizations lead to:

C1 $$\begin{aligned} \begin{array}{cc} a_1h_{i-1,j,k}^{n+1,m+1}+bh_{i,j,k}^{n+1,m+1}+c_1h_{i+1,j,k}^{n+1,m+1} +a_2h_{i,j-1,k}^{n+1,m+1}+c_2h_{i,j+1,k}^{n+1,m+1}\\ +\,a_3h_{i,j,k-1}^{n+1,m+1}+c_3h_{i,j,k+1}^{n+1,m+1}=d, \end{array} \end{aligned}$$

which is applicable to all interior nodes (grid points), and where:

C2 $$\begin{aligned} \begin{array}{l} \displaystyle a_1\displaystyle =-\frac{k_{i,j,k}^{n+1,m}+k_{i-1,j,k}^{n+1,m}}{2\Delta x_{1}^{2}}, \quad \displaystyle a_2\displaystyle =-\frac{k_{i,j,k}^{n+1,m}+k_{i,j-1,k}^{n+1,m}}{2\Delta x_2^2}, \quad a_3\displaystyle =-\frac{k_{i,j,k}^{n+1,m}+k_{i,j,k-1}^{n+1,m}}{2\Delta x_3^2}\\ \displaystyle b\displaystyle =\frac{u^{n+1,m}_{i,j,k}}{\Delta t}+\frac{k_{i+1,j,k}^{n+1,m}+2k_{i,j,k}^{n+1,m}+k_{i-1,j,k}^{n+1,m}}{2\Delta x_{1}^{2}}\\ \displaystyle \displaystyle \qquad +\,\frac{k_{i,j+1,k}^{n+1,m}+2k_{i,j,k}^{n+1,m}+k_{i,j-1,k}^{n+1,m}}{2\Delta x_{2}^{2}} +\frac{k_{i,j,k+1}^{n+1,m}+2k_{i,j,k}^{n+1,m}+k_{i,j,k-1}^{n+1,m}}{2\Delta x_{3}^{2}}\\ \displaystyle c_1=-\frac{k_{i,j,k}^{n+1,m}+k_{i+1,j,k}^{n+1,m}}{2\Delta x_{1}^{2}},\quad c_2\displaystyle =-\frac{k_{i,j,k}^{n+1,m}+k_{i,j+1,k}^{n+1,m}}{2\Delta x_2^2}, \quad \displaystyle c_3\displaystyle =-\frac{k_{i,j,k}^{n+1,m}+k_{i,j,k+1}^{n+1,m}}{2\Delta x_3^2}\\ \displaystyle d= \frac{-k_{i,j,k+1}^{n+1,m}+k_{i,j,k-1}^{n+1,m}}{2\Delta x_3}+u^{n+1,m}_{i,j,k}\frac{h_{i,j,k}^{n}}{\Delta t} \end{array} \end{aligned}$$

The CSC approximation (Rathfelder and Abriola 1994) yields $u^{n+1,m}_{i,j,k}=(\theta ^{n+1,m}_{i,j,k}-\theta ^{n}_{i,j,k})/(h_{i,j,k}^{n+1,m}-h_{i,j,k}^{n})$. In matrix form, the system of Eq. (C1) can be written as:

C3 $$\begin{aligned} \mathbf{A}(\mathbf{h}^{n+1,m})\mathbf{h}^{n+1,m+1}=\mathbf{a}(\mathbf{h}^{n+1,m}) \end{aligned}$$

where $\mathbf{h}^{n+1,m'}\in \mathbb {R}^{{k_{y}} }$ is a vector of values of $h_{i,j,k}^{n+1,m'}$, $i=1,\ldots , n_1$, $j=1,\ldots , n_2$, $k=1,\ldots , n_3$. $\mathbf{A}\in \mathbb {R}^{{k_{y}} \times {k_{y}} }$ and $\mathbf{a}\in \mathbb {R}^{{k_{y}} }$ depend only on values of the head at iteration *m*. Thus, the system (C3) is linear in $\mathbf{h}^{n+1,m+1}$. It can be solved by iterating (in *m*) within each time step *n* until convergence; that is, for each time step *n*, *m* is incremented until the residual satisfies $||\mathbf{A}(\mathbf{h}^{n+1,m+1})\mathbf{h}^{n+1,m+1}-\mathbf{a}(\mathbf{h}^{n+1,m+1})||<\varepsilon$ for some specified tolerance $\varepsilon$. In the results presented in Sect. 6.2, we use $n_1=n_2=n_3=26$ ($\Delta x_1 =\Delta x_2=\Delta x_3=0.8$ cm), $\Delta t =0.5$ s and $\varepsilon =0.01$.

## Footnotes

1 Technically the process is a random field if the index (here $\mathbf {x}$) lies in $\mathbb {R}^{L}$ where $L>1$ but the convention in the great majority of the literature is to use the term Gaussian *process* even in such cases.

2 Let $\mathbb {P}_\mathcal{X}$ be a measure on $(\mathcal{X},\mathcal{F}_\mathcal{X})$. The pushforward measure of $\mathbb {P}_\mathcal{X}$ under $\pmb {\eta }:(\mathcal{X},\mathcal{F}_\mathcal{X},\mathbb {P}_\mathcal{X})\rightarrow (\mathcal{Y},\mathcal{F}_\mathcal{Y},\mathbb {P}_\mathcal{Y})$ is defined as $\mathbb {P}_\mathcal{Y}(F)=\mathbb {P}_\mathcal{X}\circ \pmb {\eta }^{-1}(F)$ for $F\in \mathcal{F}_\mathcal{Y}$. We characterize the measures by their probability density functions (pdfs) with respect to Lebesgue measure. In this work a Gaussian distribution is placed on the inputs.

3 See http://subsurface.gr/joomla/msim_doc/twoD_examples_help.html for full details of the implementation, including the domain, mesh generation and boundary conditions. Last accessed 29 August 2017.

4 If $Z(\mathbf {x})$ has a mean and variance of $\mu$ and $\nu$, then the mean and variance of the log-normal process $\exp (Z(\mathbf {x}))$ are $\mu '=\exp (\mu +\nu /2)$ and $\nu '=\exp (2\mu +\nu )(\exp (\nu )-1)$, respectively.
